# Exploring the Anti-Influenza Activity of *closo*-Borate Platforms: Structure–Activity Relationship of Amino Acid-Functionalized *closo*-Dodecaborate Derivatives Against Influenza Virus A/Cheboksary/125/2020 (H1N1)pdm09

**DOI:** 10.3390/molecules30214225

**Published:** 2025-10-29

**Authors:** Timur M. Garaev, Ilya I. Yudin, Natalya V. Breslav, Tatyana V. Grebennikova, Evgenii Yu. Matveev, Elizaveta A. Eshtukova-Shcheglova, Ilya E. Sokolov, Varvara V. Avdeeva, Konstantin Yu. Zhizhin, Nikolai T. Kuznetsov

**Affiliations:** 1 Gamaleya National Research Center for Epidemiology and Microbiology, Ministry of Health of Russian Federation, Moscow 123098, Russia; tmgaraev@gmail.com (T.M.G.);; 2 Kurnakov Institute of General and Inorganic Chemistry, Russian Academy of Sciences, Moscow 119991, Russia; cat1983@yandex.ru (E.Y.M.);; 3 Institute of Fine Chemical Technologies named after M. V. Lomonosov, MIREA—Russian Technological University, Moscow 119571, Russia; 4 Federal Research Centre of Nutrition and Biotechnology, Moscow 109240, Russia

**Keywords:** antiviral activity, viroporins, amino acids, polyhedral boron anions, *closo*-dodecaborate anion, tetrahydropyran, opening of cyclic substituents

## Abstract

The emergence of drug-resistant influenza virus strains necessitates the development of novel antiviral agents with unique mechanisms of action. This study presents the synthesis and in vitro evaluation of a new class of antiviral compounds: sodium salts of amino acid ester conjugates based on the *closo*-dodecaborate anion [B_12_H_12_]^2−^, linked via a tetrahydropyran-derived spacer (Na_2_[B_12_H_11_O(CH_2_)_6_C(O)X], where X = L-Trp-OMe (Na_2_**2**); L-His-OMe (Na_2_**3**); L-Met-OMe (Na_2_**4**); Pld-OMe (Na_2_**5**)). The antiviral activity was assessed against contemporary, multidrug-resistant influenza A virus strains, including A/Cheboksary/125/2020 (H1N1)pdm09 and A/IIV-Orenburg/83/2012 (H1N1)pdm09. Cross-platform comparison revealed that the dodecaborate-tryptophan conjugate Na_2_**2** exhibited comparable efficacy to its lead decaborate analog against the Orenburg strain while demonstrating potent activity (IC_50_ = 5.0 µg/mL) against the Cheboksary strain with reduced susceptibility to neuraminidase inhibitors (oseltamivir; zanamivir) and complete resistance to M2 channel blockers. The histidine-based conjugate Na_2_**3** also showed significant efficacy against the Cheboksary strain, while methionine and lactam derivatives (Na_2_**4**; Na_2_**5**) remained inactive. This work confirms boron clusters as versatile platforms for antiviral development and establishes structure–activity relationships crucial for optimizing both B_10_ and B_12_-based therapeutics against resistant influenza strains.

## 1. Introduction

The COVID-19 pandemic, declared by the WHO on 11 March 2020, created unprecedented global disruption across all societal sectors [1,2,3]. While the public health emergency was lifted in May 2023, concerns remain about potential SARS-CoV-2 variants [4,5]. The post-pandemic period has witnessed resurgent influenza activity, particularly A(H1N1)pdm09, alongside persistent challenges in influenza management [6,7]. Annual influenza continues to cause significant global burden with 3–5 million severe cases and 290,000–650,000 deaths annually [8,9], while co-infections with SARS-CoV-2 demonstrate increased morbidity and mortality [10,11].

Current influenza control strategies face substantial limitations. Vaccines, while effective, require annual reformulation due to viral antigenic drift [12,13], and antiviral options are increasingly compromised by resistance. Over 90% of circulating strains resist M2 blockers [14,15,16,17], while neuraminidase inhibitors face emerging resistance mutations like H274Y and I117T [18]. This therapeutic gap necessitates novel antiviral approaches with distinct mechanisms of action [19].

Polyhedral boron clusters [B*_n_*H*_n_*]^2–^ (*n* = 10,12) represent a promising platform for drug design [20,21,22,23,24,25,26,27,28,29]. These clusters can be selectively functionalized via electrophile-induced nucleophilic substitution (EINS) using Lewis acids or carbocations [30,31,32,33], allowing for the synthesis of a wide range of derivatives [34,35,36,37,38,39]. Of specific interest are derivatives containing tetrahydrofuran (THF) substituents, which serve as versatile precursors for further modification with biologically active groups, such as amino acids, via an alkoxy spacer [40,41,42,43,44,45,46].

The proven antiviral efficacy of membrane-tropic carbocyclic amino acid and peptide derivatives against influenza A, hepatitis C, and coronaviruses [47] provides a strong rationale for developing new classes of inorganic–organic hybrids based on boron clusters.

We hypothesize that the *closo*-dodecaborate [B_12_H_12_]^2−^ anion represents a versatile platform for developing novel membrane-tropic antiviral agents against influenza. We propose that functionalization of this inorganic core with tetrahydrofuran spacers and selected amino acid residues (Trp, His, Met, Pld) will yield compounds with enhanced antiviral potency against contemporary influenza strains while maintaining low cytotoxicity profiles. Furthermore, we anticipate that the larger B_12_ cluster will confer distinct biological properties compared to previously studied B_10_ analogs, potentially overcoming limitations of current therapeutics through a unique mechanism of action.

This hypothesis is grounded in our previous success with *closo*-decaborate derivatives demonstrating significant activity against influenza A/IIV-Orenburg/83/2012 (H1N1)pdm09 [48,49,50,51]. The strategic selection of the contemporary A/Cheboksary/125/2020 (H1N1)pdm09 strain for evaluation ensures clinical relevance in the current epidemiological landscape.

The aim of this work is to synthesize and characterize a novel series of Na_2_[B_12_H_11_O(CH_2_)_6_X] compounds and evaluate their antiviral potential against this relevant influenza strain, thereby testing our central hypothesis that boron cluster–amino acid hybrids represent a promising new class of anti-influenza agents.

## 2. Results and Discussion

### 2.1. Syntheses

**Scheme 1:**
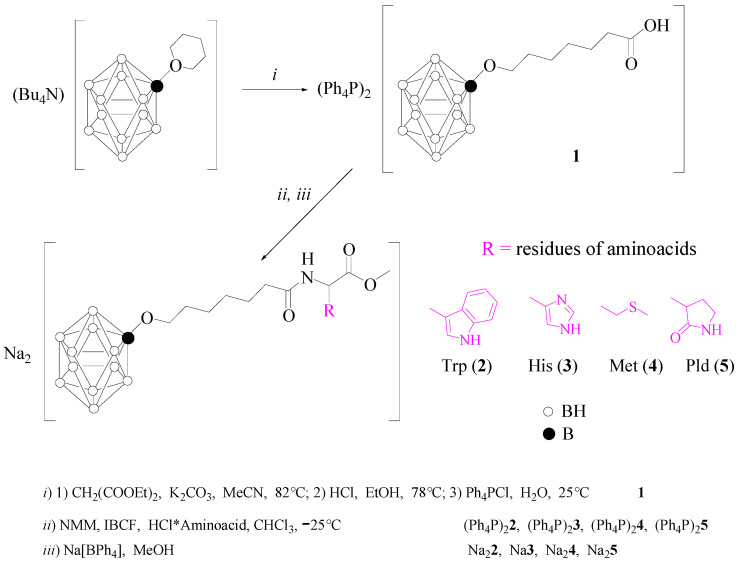
Synthesis of sodium salts of amino acid derivatives of the *closo*-dodecaborate anion Na_2_**2**, Na_2_**3**, Na_2_**4**, and Na_2_**5** conjugated to amino acid (Trp, His, Met, Pld) esters via a linker derived from the ring-opening of a tetrahydrofuran molecule via the COOH precursor (Ph_4_P)_2_**1**.

In this work, a series of novel compounds based on the *closo*-dodecaborate anion [B_12_H_12_]^2−^, chemically linked to methyl ester residues of the amino acids tryptophan, histidine, and methionine and a synthetic amino acid analog with a butyrolactam (pyrrolidin-2-one) side chain, were synthesized. The synthesis commenced with the modification of the boron cluster in the (n-Bu_4_N)_2_[B_12_H_12_] salt with tetrahydropyran to form the (*n*-Bu_4_N)[B_12_H_11_OC_5_H_10_] derivative. This was followed by ring-opening of the oxonium cyclic substituent and hydrolysis of an alkyl malonate followed by thermal decarboxylation, yielding 2-(6-carboxyhexoxy)undecahydro-*closo*-dodecaborate tetraphenylphosphonium salt (Ph_4_P)_2_**1** (analogous to the method described for the *closo*-decaborate derivatives [51]). Subsequently, condensation with the corresponding amino acid methyl ester was performed using peptide synthesis methods (mixed anhydride method), as outlined in Scheme 1. This procedure afforded the target compounds as tetraphenylphosphonium salts, [B_12_H_11_O(CH_2_)_6_C(O)X]^2−^, where X = Trp-OMe (**2**), His-OMe (**3**), Met-OMe (**4**), Pld-OMe (**5**), which were then converted into the corresponding sodium salts Na_2_**2**–Na_2_**5** (Scheme 1).

### 2.2. Study of the Cytotoxic Activity of Compounds Na_2_**2**–Na_2_**5** on MDCK Cell Culture

**Figure 1:**
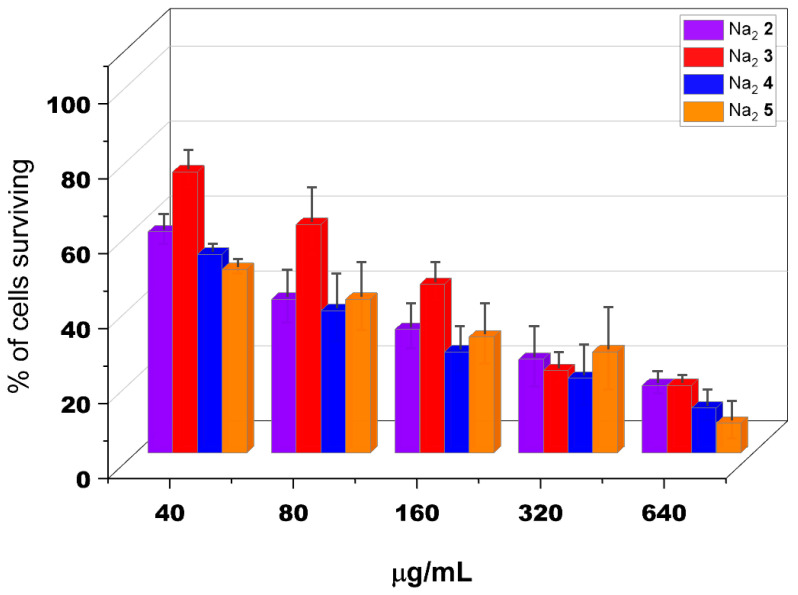
Toxicity of compounds Na_2_**2**–Na_2_**5** to MDCK cell culture (*n* = 5).

The investigation of the cytotoxic effects of compounds Na_2_**2**–Na_2_**5** on MDCK cell monolayers was conducted according to a previously described methodology [51]. Five compound concentrations (40–640 μg/mL) were tested. The compounds were applied to fully confluent monolayers (100% confluency) and incubated for 48 h at 37 °C in a thermostat. Cell viability was assessed using a vital dye followed by optical density (OD) measurements in each well of a 96-well plate. The OD values were compared to those of control wells (without compounds) to calculate the percentage of viable cells. Figure 1 presents the results as a bar chart.

The diagram clearly shows that the compound containing the L-histidine amino acid ester moiety (Na_2_**3**) exhibited the lowest cytotoxicity against MDCK cell monolayers, with a CC_50_ value of 160 μg/mL. The other compounds (Na_2_**2**, Na_2_**4**, Na_2_**5**) demonstrated higher cytotoxicity, with CC_50_ values of 80 μg/mL.

### 2.3. Study of the Antiviral Activity of Synthesized Compounds Na_2_**2**–Na_2_**5** Against A/Cheboksary/125/2020 (H1N1)pdm09

Evaluation of the antiviral activity of compounds Na_2_**2**–Na_2_**5** was performed in an in vitro assay using MDCK cell lines. The test compounds and the virus were introduced simultaneously to a confluent cell monolayer. The experiment was conducted in 96-well immunological plates, followed by a cell-based ELISA to quantify the percentage inhibition of viral replication, as previously described by our group [51]. The compounds were tested at six concentrations ranging from 0.5 to 10.0 μg/mL.

The compounds were screened against a recently isolated strain of influenza virus, A/Cheboksary/125/2020 (H1N1)pdm09, obtained in 2020 from a patient with pneumonia. This strain exhibited reduced susceptibility to both oseltamivir (IC_50_ = 98.9 nM) and zanamivir (IC_50_ = 13.1 nM) [52]. Furthermore, this influenza A strain was fully resistant to M2 ion channel blockers (rimantadine and amantadine), i.e., the strain contained the marker substitution S13N [53].

**Figure 2:**
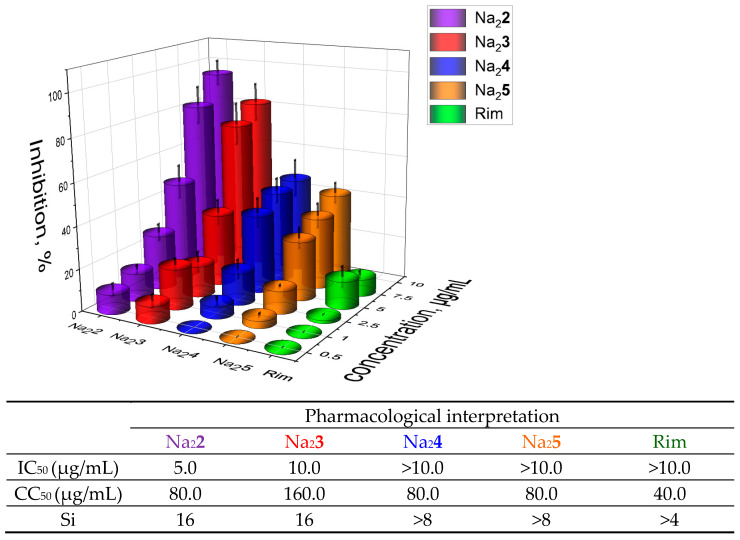
Antiviral activity of compounds Na_2_**2**–Na_2_**5** against the influenza A virus strain A/Cheboksary/125/2020 (H1N1)pdm09.

Figure 2 summarizes the mean values of the antiviral activity results from independent experiments (four replicates in three independent trials) conducted under identical conditions. The results are presented with the standard deviation (SD).

As illustrated in Figure 2, the compounds exhibited varying degrees of activity against the virus under the therapeutic-prophylactic administration protocol. Among the tested compounds, Na_2_**2**, which contains an L-tryptophan residue, demonstrated the most potent antiviral effect (see Figure 2). This compound achieved an IC_50_ value at a concentration of 5.0 μg/mL.

A slightly reduced yet significant effect was observed for compound Na_2_**3**, functionalized with an imidazole ring from an L-histidine residue. At the same time, it should be noted that due to the somewhat lower toxic effect of Na_2_**3** compared to the other tested derivatives, compound Na_2_**3** demonstrates a selectivity index (Si) of 16, similar to compound Na_2_**2**. In contrast, compounds Na_2_**4** and Na_2_**5**, featuring a methyl sulfide fragment and a lactam group, respectively, did not reach the IC_50_ threshold within the tested concentration range. It is plausible that their half-maximal inhibitory concentration may lie outside the scope of this experimental design.

**Figure 3:**
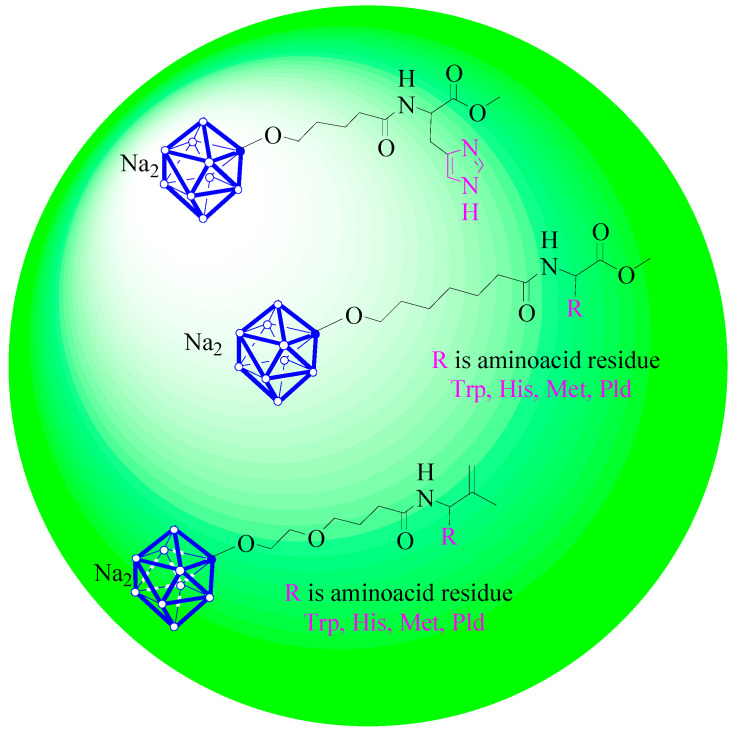
Structures of known B_10_-based compounds found to be active against A/IIV-Orenburg/83/2012 (H1N1)pdm09 virus [48,49,50,51].

**Figure 4:**
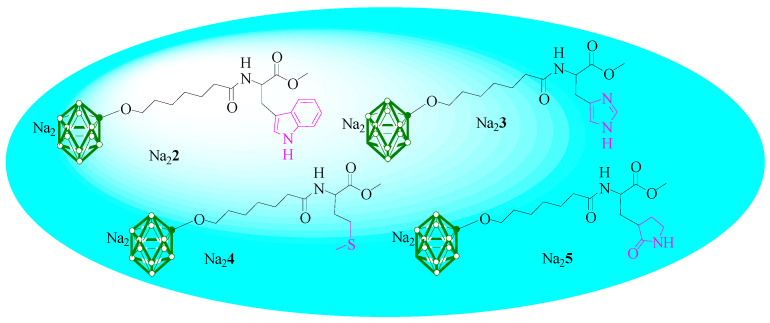
Structures of new B_12_-based compounds Na_2_**2**–Na_2_**5** synthesized in this work.

This study extends our ongoing research into the anti-influenza properties of boron cluster anions conjugated with amino acid residues. Previous work has established the activity of *closo*-decaborate derivatives featuring tetrahydrofuran or dioxane spacers coupled with a similar set of amino acids [48,49,50,51]. Figure 3 and Figure 4 present the structural formulas of the previously studied B_10_-based derivatives, alongside the novel B_12_-based series, where R represents the functional group of the respective amino acid residue.

This apparent potency difference suggests that the smaller, more sterically constrained B_10_ core may be more favorable for the intended biological interaction, potentially due to its distinct geometry and electronic properties compared to the larger B_12_ cluster.

During the study of the cytotoxic properties of the *closo*-dodecaborate derivatives with L-amino acid ester residues, a slightly greater inhibitory effect on the growth of the cell monolayer was revealed, which manifested itself in changes in cell morphology. This was noted during the examination of the cell monolayer under a microscope. This did not lead to the death of the cell monolayer or a delay in its growth at concentrations of 40 and 80 µg/mL, as shown using a vital dye; however, an abnormal geometry of the cell monolayer was observed.

Another observation when working with derivatives Na_2_**2**–Na_2_**5** was their somewhat lower ability to dissolve in water or aqueous solutions (e.g., cell culture medium) compared to B_10_-based analogs. Reduced water solubility under in vitro experimental conditions means lower bioavailability of the substance and sometimes undesirable side effects associated with toxic action.

A direct and quantitative comparison of efficacy between the B_10_ and B_12_ series is complicated by a critical methodological difference: the two compound classes were evaluated against different influenza virus strains. The previously reported B_10_ derivatives demonstrated high activity against the A/IIV-Orenburg/83/2012 (H1N1)pdm09 strain, whereas the present study assessed the B_12_ derivatives against the more recent A/Cheboksary/125/2020 (H1N1)pdm09 isolate.

### 2.4. Study of the Antiviral Activity of Na_2_2 Against A/IIV-Orenburg/83/2012

The selection of the A/Cheboksary/125/2020 strain for this study is strategically significant. While the A/IIV-Orenburg/83/2012 strain provided initial proof-of-concept, the Cheboksary strain represents a more recent and relevant circulating variant. Using a con-temporary clinical isolate ensures that the evaluated antiviral efficacy is directly relevant to the current epidemiological landscape, providing a more accurate and reliable assess-ment of the compounds’ potential for therapeutic application.

The presumed protein target of the proposed compounds is the proton-conducting M2 channel of influenza A virus. The study examined the M gene sequences of both strains (A/IIV-Orenburg/83/2012|M2|EPI_ISL_1360030 and A/Human/Cheboksary/125/2020(H1N1)|M2|EPI_ISL_483726), isolated and characterized at the Laboratory of Etiology and Epidemiology of Influenza at the Gamaleya National Research Center for Epidemiology and Microbiology of the Ministry of Health of Russian Federation. Particular attention was paid to the M2 sequences, specifically the transmembrane domain (TM-domain) responsible for proton conductance into the virion, which is of interest for inhibitor binding. Although the surface of the M2 TM-domain primarily consists of hydrophobic residues, amino acid substitutions are known and can alter the surface within the channel pore. The analysis revealed a small number of amino acid substitutions in the M gene, with only one in the entrance group of the TM-domain: S23N. Furthermore, the residue at position 23 is located on the outer side of the α-helix turn and does not significantly affect the internal space of the M2 channel pore (see Supplementary Information, Figure S1).

**Figure 5:**
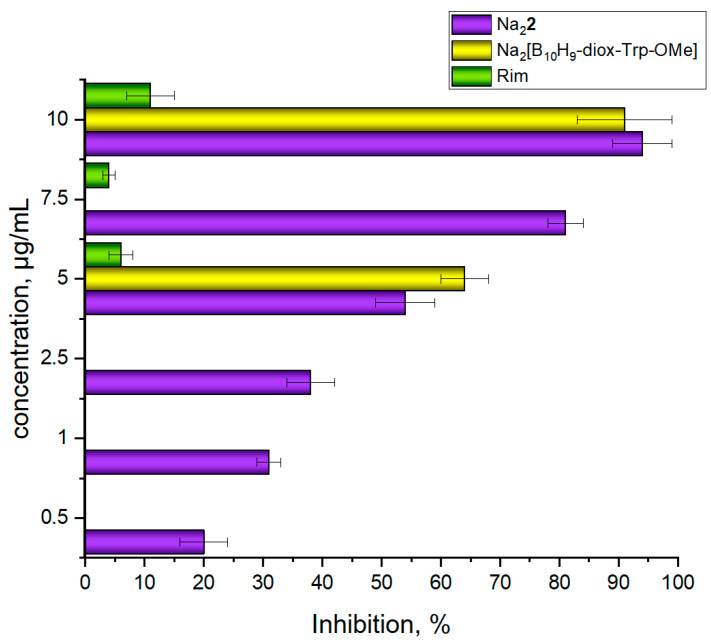
In vitro comparison of the antiviral properties of Na_2_**2** and rimantadine hydrochloride with the data reported for Na_2_[B_10_H_9_–O(CH_2_)_2_O(CH_2_)_3_C(O)–Trp–OCH_3_] [51] against influenza virus strain A/IIV-Orenburg/83/2012, with simultaneous administration of compound and virus.

For a correct comparison of the antiviral properties of the B_10_ and B_12_ platforms, a study of the B_12_ platform’s lead compound, Na_2_**2**, was conducted against the seasonal strain A/IIV-Orenburg/83/2012. The results were compared with literature data for the B_10_ platform’s lead compound Na_2_[B_10_H_9_–O(CH_2_)_2_O(CH_2)3_C(O)–Trp–OCH_3_] [51] and rimantadine hydrochloride. The data are presented in Figure 5.

The Cheboksary strain is of particular interest as it is a contemporary clinical variant characterized by reduced susceptibility to standard-of-care neuraminidase inhibitors (oseltamivir and zanamivir) and full resistance to M2 ion channel blockers. Consequently, the observed activity of compounds Na_2_**2** and Na_2_**3**, even if ostensibly lower than that of their B_10_ analogues, remains highly significant. It confirms that the boron cluster-amino acid conjugate platform retains efficacy against a currently circulating, drug-resistant influenza strain, underscoring its potential as a promising strategy for overcoming antiviral resistance.

Thus, while the B_10_ architecture may offer an intrinsic advantage, the biological activity is also demonstrably influenced by the specific viral target. The consistent structure–activity relationship across both series—with tryptophan and histidine derivatives showing superior activity—provides valuable insights for the rational design of next-generation antiviral agents based on boron clusters.

### 2.5. Prediction of SAR Molecular Properties for Na_2_**2** and Na_2_[B_10_H_9_–O(CH_2_)_2_O(CH_2_)_3_C(O)–Trp–OCH_3_]

During early drug development stages, structure–activity relationship (SAR) services based on large empirical datasets are frequently employed. The proposed lead compounds Na_2_**2** and Na_2_[B_10_H_9_–O(CH_2_)_2_O(CH_2_)_3_C(O)–Trp–OCH_3_] reported [51] were evaluated for key molecular properties including solubility, biological activity, and toxicity using the online admetSAR service [54]. Absorption, distribution, metabolism, excretion, and toxicity (ADMET) properties are particularly crucial in drug development (see Supplementary Information, Table S1).

A compound’s aqueous solubility significantly influences its absorption and distribution characteristics in both in vitro and in vivo experiments. SAR analysis provides LogS values—logarithmic measures of water solubility characterized as: log S ≥ 0 (Highly soluble); −2 ≤ log S < 0 (Soluble); −4 ≤ log S < −2 (Slightly soluble); log S < −4 (Insoluble).

Our in vitro studies demonstrated that Na_2_**2** exhibited somewhat lower solubility compared to Na_2_[B_10_H_9_–O(CH_2_)_2_O(CH_2_)_3_C(O)–Trp–OCH_3_], consistent with admetSAR predictions: −3.3285 mol/L versus −2.8189 mol/L.

Another challenge in achieving favorable pharmaceutical profiles was the observed morphological changes in MDCK cells treated with B_12_ platform compounds, particularly Na_2_**2**. Comparing membrane permeability parameters, Na_2_**2** showed reduced Caco-2 cell permeability (LogP_app_ = 0.4415) versus the B_10_ platform compound (−0.2584). These differences may be attributed not only to the B_12_ platform itself but also to its alkoxy spacer (derived from tetrahydrofuran) compared to the oxygen-containing spacer (derived from dioxane) in the *closo*-dodecaborate-based compound.

## 3. Materials and Methods

### 3.1. Materials 

All chemicals were commercially purchased and used without further purification. These included hydrochlorides of methyl esters of L-amino acids (histidine (His), tryptophan (Trp), methionine (Met), Ala(2-pyrrolidone) (Pld)) (95%, Sigma-Aldrich, St. Louis, MO, USA), tetrahydropyran (99%, Sigma-Aldrich, St. Louis, MO, USA), malonic ester (98%, Sigma-Aldrich, St. Louis, MO, USA), acetonitrile (99%, Sigma-Aldrich, St. Louis, MO, USA), potassium carbonate (reagent grade, Khimmed, Moscow, Russia), hydrochloric acid (36%, reagent grade, Khimmed, Moscow, Russia), N-methylmorpholine (NMM) (99%, Sigma-Aldrich, St. Louis, MO, USA), *iso*-butyl chloroformate (IBCF) (99%, Sigma-Aldrich, St. Louis, MO, USA), tetraphenylphosphonium chloride (99%, Sigma-Aldrich, St. Louis, MO, USA), and sodium tetraphenylborate (99.5%, Sigma-Aldrich, St. Louis, MO, USA).

Tetrabutylammonium [2-(1-(tetrahydropyranyl))]undecahydro-*closo*-decaborate (*n*-Bu_4_N)[B_12_H_11_O(CH_2_)_5_] was synthesized according to a previously developed procedure [55].

### 3.2. Virological Tests

Virological evaluation was performed using the pandemic influenza A virus strains A/IIV-Orenburg/83/2012(H1N1)pdm09 and A/Cheboksary/125/2020 (H1N1)pdm09. This strain used Gamaleya National Research Center for Epidemiology and Microbiology, Ministry of Health of Russian Federation exhibits documented resistance to rimantadine and amantadine antiviral agents.

Infectious titer determination for the influenza virus was conducted in MDCK (NBL-2) continuous cell line (ATCC CCL-34, American Type Culture Collection), following established virological methodologies.

### 3.3. Physicochemical Characterization Methods

The synthesized compounds were characterized using a suite of analytical techniques. Fourier-transform infrared (FTIR) spectra were recorded on an Infralum FT-02 spectrometer (Lumex, St. Petersburg, Russia) in the range of 400–4000 cm^−1^ using KBr pellets. 

Nuclear magnetic resonance (NMR) spectra (^1^H, ^11^B, ^13^C) were acquired on a Bruker DPX-300 spectrometer (Bruker, Billerica, MA, USA) operating at frequencies of 300.3, 96.32, and 75.49 MHz, respectively. Samples were dissolved in DMSO-d_6_, and the deuterium signal of the solvent was used for internal locking. NMR spectroscopy data for compounds are present in Supplementary Information.

Elemental analysis for boron was performed on an ELAN DRC-e PerkinElmer inductively coupled plasma mass spectrometer (PerkinElmer Inc., Shelton, CT, USA). The content of carbon, hydrogen, and nitrogen in the samples was determined on a Eurovector EuroEA 3000 CHNS elemental analyzer (Eurovector Instruments, Milan, Italy). 

Mass spectrometric analysis was performed using an Agilent 1200 series HPLC system (G1311A quaternary pump) (Agilent Technologies, Santa Clara, CA, USA) coupled to a triple quadrupole mass spectrometer (TSQ Quantum Access MAX). Mass-spectroscopy data for compounds are present in Supplementary Information. 

Tetraphenylphosphonium 2-(6-carboxyhexoxy)undecahydro-*closo*-dodecaborate, (Ph_4_P)_2_[B_12_H_11_O(CH_2_)_6_COOH], (Ph_4_P)_2_**1**. (*n*-Bu_4_N)[B_12_H_11_O(CH_2_)_5_] (1.05 g, 2.24 mmol), diethyl malonate (1.02 mL, 6.9 mmol), and potassium carbonate (1.54 g, 11.2 mmol) were combined in acetonitrile (50 mL). The resulting suspension was stirred and heated under reflux for 2 h. After cooling, the mixture was filtered to remove the excess potassium carbonate. The pale yellow filtrate was concentrated to a small volume of a yellow, viscous liquid. This residue was treated with a solution of 11% hydrochloric acid (70 mL) in ethanol (15 mL). The resulting solution was heated under reflux for 24 h. The cooled, transparent mixture was then evaporated to dryness. The white solid obtained was dissolved in water (10 mL), and a solution of Ph_4_PCl (1.68 g, 4.48 mmol) in water (15 mL) was added. The resulting white precipitate was collected by filtration and dried under high vacuum at 60 °C for 2 h, yielding 1.71 g (79%) of the product.

^1^H NMR (DMSO-d_6_, δ, ppm): −0.50–0.50 (11H, m), 1.19–1.27 (4H, m), 1.31 (4H, m), 2.16 (2H, t, *J* = 7.2 Hz), 3.22 (2H, m). ^11^B {^1^H} NMR (DMSO-d_6_, δ, ppm): 8.9 (s, B(1)), −14.3 (s, B(2–6)), −15.9 (s, B(7–11)), −20.7 (s, B(12)). ^13^C NMR (DMSO-d_6_, δ, ppm): 25.2; 26.2; 29.3; 34.3; 52.1, 68.5; 171.4. IR (KBr, cm^−1^): 3442 (ν(O-H)), 2459 (ν(B-H)), 1692 ((ν(C=O)). Found, %: B 13.26, C 68.17, H 6.63. Calculated, %: B 13.45, C 68.47, H 6.69. ESI MS. Found, *m*/*z*: 625.59 {Ph_4_P^+^ + [B_12_H_11_O(CH_2_)_6_COOH]^2−^}. (C_31_H_44_B_12_O_3_P). Calculated: M = 625.38.

Tetraphenylphosphonium methyl-2-[2-(2-(2-carbonyl)amino]-3-(1H-indol-2-yl)propanoate)ethoxy)ethoxy]undecahydro-*closo*-dodecaborate (Ph_4_P)_2_[B_12_H_11_O(CH_2_)_5_C(O)-Trp-OCH_3_] (Ph_4_P)_2_**2**. A weighted sample of (Ph_4_P)_2_[B_12_H_11_O(CH_2_)_5_COOH] (0.3 g, 0.31 mmol) and 0.034 mL (0.31 mmol) N-methylmorpholine (NMM) were dissolved in 10 mL CHCl_3_ and cooled to –25 °C. With stirring, 0.041 mL (0.31 mmol) isobutyl chloroformate (IBCF) was added. The mixture was stirred at −25 °C for 10 min to form the mixed anhydride. Then a pre-cooled (−20 to −25 °C) solution of 0.079 g (0.31 mmol) HCl·H-Trp-OMe and 0.034 mL (0.31 mmol) NMM was added. The reaction mixture was stirred for 1 h at −20 to −15 °C, then 1 h at 0 °C, and left for 18 h while warming to 24 °C. The mixture was then washed sequentially with H_2_O (10.0 mL × 1), 0.5 N H_2_SO_4_ (4.0 mL × 1), 0.5 N KHCO_3_ (10.0 mL × 2), and H_2_O again (5.0 mL × 1). The organic layer was separated, dried over anhydrous Na_2_SO_4_, and concentrated on a rotary evaporator (45 °C, 15 mmHg) to give an oily product that solidified upon standing. Yield, 0.354 g (97.8%).

^1^H NMR (DMSO-d_6_, δ, ppm): 0.50—−0.50 (m, 11H), 1.08–1.23 (6H, m); 1.91 (2H, t, *J* = 6.9 Hz); 3.16 (2H, m); 3.22–3.39 (4H, m); 3.60 (3H, s); 4.49 (m, 1H); 6.63 (br. s, 1H), 6.95 (dd, *J* = 8.6, 7.4 Hz, 1H), 7.06 (dd, *J* = 8.9, 7.4 Hz, 1H), 7.15 (s, 1H), 7.32 (d, *J* = 8.6 Hz, 1H), 7.46 (d, *J* = 8.9 Hz, 1H), 7.69–8.03 (m, 40H), 10.91 (s, 1H). ^11^B {^1^H} NMR (DMSO-d_6_, δ, ppm): 6.3 (s, B(1)), −16.9 (s, B(2–6)), −18.4 (s, B(7–11)), −21.3 (s, B(12)). ^13^C NMR (DMSO-d_6_, δ, ppm): 25.4; 26.2; 28.6; 31.5; 41.5; 42.3; 51.6; 53.2; 67.7; 111.5–136.1; 169.4, 172.0. IR (KBr, cm^−1^): 2451 (ν(B-H)), 1720 ((ν(C=O)). Found, %: B 11.01, C 69.40, H 5.72, N 2.39. Calculated, %: B 11.23, C 69.67, H 5.76, N 2.43. ESI MS. Found, *m*/*z*: 825.71 {Ph_4_P^+^ + [B_12_H_11_O(CH_2_)_6_COTrp]^2−^}. (C_43_H_56_B_12_N_2_O_4_P). Calculated: M = 825.62. Found, *m*/*z*: 625.63 {Ph_4_P^+^ + [B_12_H_11_O(CH_2_)_6_COOH]^2−^}. (C_31_H_44_B_12_O_3_P). Calculated: M = 625.38.

Sodium methyl-2-[2-(2-(2-carbonyl)amino]-3-(1H-indol-2-yl)propanoate)ethoxy)ethoxy]undecahydro-*closo*-dodecaborate Na_2_**2**. The product (Ph_4_P)_2_**2** obtained is subjected to counterion exchange from Ph_4_P^+^ to Na^+^ using the following method. The tetraphenylphosphonium salt (Ph_4_P)_2_[B_12_H_11_O(CH_2_)_6_COTrp] (0.233 g, 0.20 mmol) is dissolved in a minimal volume of methanol (2 mL). A saturated aqueous solution of sodium tetraphenylborate Na[BPh_4_] (0.137 g, 0.40 mmol) is then added with vigorous stirring. The immediate formation of a dense white precipitate of tetraphenylphosphonium tetraphenylborate (Ph_4_P BPh_4_) indicates the progress of the metathesis reaction. The mixture is stirred for an additional 1–2 h at room temperature to ensure complete precipitation. The insoluble precipitate is then removed by filtration. The filtrate is concentrated under reduced pressure to isolate the sodium salt of the product, which is typically obtained as a white solid. The product is then dried under high vacuum. Yield, 0.058 g (54%).

Tetraphenylphosphonium methyl-2-[2-(2-(2-carbonyl)amino]-3-(1*H*-imidazol-4-yl)propanoate)ethoxy)ethoxy]undecahydro-*closo*-dodecaborate (Ph_4_P)_2_[B_12_H_11_O(CH_2_)_5_C(O)-His-OCH_3_] (Ph_4_P)_2_**3** was obtained similarly to (Ph_4_P)_2_**2** starting from 0.3 g (0.31 µm) (Ph_4_P)_2_[B_12_H_11_O(CH_2_)_5_COOH] and 0.075 g (0.31 µm) 2HCl*H-His-OMe. Yield, 0.289 r (83.2%), oily.

^1^H NMR (DMSO-d_6_, δ, ppm): 0.50—−0.50 (m, 11H),), 1.08–1.23 (6H, m); 1.91 (2H, s); 3.22 (2H, m); 3.39 (4H, m); 3.94 (3H, s); 4.27 (m, 1H); 6.64 (br. s, 1H), 7.36 (s, 1H), 7.70–8.02 (m, 40H), 8.27 (s, 1H), 8.96 (br. s, 1H). ^13^C NMR (DMSO-d_6_, δ, ppm): 25.2, 26.1, 28.6, 32.2, 42.0, 56.5, 66.4, 72.9, 117.6, 118.7, 131.0, 135.1, 135.9, 168.7, 174.8. ^11^B {^1^H} NMR (DMSO-d_6_, δ, ppm): 6.3 (s, B(1)), −16.8 (s, B(2–6)), −18.4 (s, B(7–11)), −21.2 (s, B(12)). IR (KBr, cm^−1^): 2447 (ν(B-H)), 1708 ((ν(C=O)). Found, %: B 11.45, C 66.46, H 6.53, N 3.74. Calculated, %: B 11.63, C 66.73, H 6.59, N 3.77. ESI MS. Found, *m*/*z*: 776.69 {Ph_4_P^+^ + [B_12_H_11_O(CH_2_)_6_COHis]^2−^}. (C_38_H_53_B_12_N_3_O_4_P). Calculated: M = 776.55. Found, *m*/*z*: 625.65 {Ph_4_P^+^ + [B_12_H_11_O(CH_2_)_6_COOH]^2−^}. (C_31_H_44_B_12_O_3_P). Calculated: M = 625.38.

Sodium methyl 2-[2-(2-{2-[(3-(1H-imidazol-4-yl)-1-methoxy-1-oxopropan-2-yl)amino]-2-oxoethoxy}ethoxy)ethoxy]undecahydro-*closo*-dodecaborate Na_2_[B_12_H_11_O(CH_2_)_6_COHis-OMe] (Na_2_**3**). The procedure is analogous, as indicated for Na_2_**2**. (Ph_4_P)_2_[B_12_H_11_O(CH_2_)_6_COHis] (0.220 g, 0.20 mmol) was dissolved in methanol (2 mL). A solution of Na[BPh_4_] (0.137 g, 0.40 mmol) in methanol (2 mL) was added, resulting in the formation of a white precipitate. The precipitate was removed by filtration. The filtrate was concentrated under reduced pressure to afford a white hygroscopic powder, which was dried under high vacuum. Yield, 0.049 g (51%).

Tetraphenylphosphonium methyl 2-[2-(2-(2-carbonyl)amino]-4-(methylsulfanyl)butanoate)ethoxy)ethoxy]undecahydro-*closo*-dodecaborate (Ph_4_P)_2_[B_12_H_11_O(CH_2_)_5_C(O)-Met-OCH_3_], (Ph_4_P)_2_**4** was prepared similarly to (Ph_4_P)_2_**2**, starting from 0.3 g (0.31 μM) (Ph_4_P)_2_[B_12_H_11_O(CH_2_)_5_COOH] and 0.062 g (0.31 μM) HCl*H-Met-OMe. Yield, 0.312 g (90.4%), oily.

^1^H NMR (DMSO-d_6_, δ, ppm): 0.50—−0.50 (m, 11H),), 1.11–1.23 (6H, m); 1.91 (2H, s); 2.24 (3H, s); 3.37 (2H, m); 3.66 (4H, m); 4.27 (m, 1H); 6.64 (br. s, 1H), 7.70–8.02 (m, 40H). ^13^C NMR (DMSO-d_6_, δ, ppm): 23.5, 29.0, 33.4, 35.4, 39.1, 40.8; 52.4, 56.5, 68.2, 117.6, 118.7, 130.9, 135.1, 135.9, 167.4, 172.2. ^11^B {^1^H} NMR (DMSO-d_6_, δ, ppm): 5.7 (s, B(1)), −16.7 (s, B(2–6)), −18.1 (s, B(7–11)), −21.3 (s, B(12)). IR (KBr, cm^−1^): 2448 (ν(B-H)), 1718 ((ν(C=O)). Found, %: B 11.39, C 64.82, H 6.65, N 1.21. Calculated, %: B 11.52, C 65.07, H 6.71, N 1.24. ESI MS. Found, *m*/*z*: 786.65 {Ph_4_P^+^ + [B_12_H_11_O(CH_2_)_6_COMet]^2−^}. (C_37_H_55_B_12_NO_5_PS). Calculated: M = 786.60. Found, *m*/*z*: 625.60 {Ph_4_P^+^ + [B_12_H_11_O(CH_2_)_6_COOH]^2−^}. (C_31_H_44_B_12_O_3_P). Calculated: M = 625.38.

Sodium methyl-2-[2-(2-(2-carbonyl)amino]-4-(methylsulfanyl)butanoate)ethoxy)ethoxy]undecahydro-*closo*-dodecaborate Na_2_[B_12_H_11_O(CH_2_)_6_COMet-OMe] (Na_2_**4**). (Ph_4_P)_2_**4** (0.222 g, 0.20 mmol) was dissolved in methanol (2 mL). A solution of Na[BPh_4_] (0.137 g, 0.40 mmol) in methanol (2 mL) was added, resulting in the formation of a white precipitate. The precipitate was removed by filtration. The filtrate was concentrated under reduced pressure to afford a white hygroscopic powder, which was dried under high vacuum. Yield, 0.052 g (54%).

Tetraphenylphosphonium methyl 2-[2-(2-(2-carbonyl)amino]-3-(2-oxopyrrolidin-3-yl)propanoate)ethoxy)ethoxy]undecahydro-*closo*-dodecaborate (Ph_4_P)_2_[B_12_H_11_O(CH_2_)_5_C(O)-Pld-OCH_3_], (Ph_4_P)_2_**5** was prepared similarly to (Ph_4_P)_2_**2**, starting from 0.3 g (0.31 μM) (Ph_4_P)_2_[B_12_H_11_O(CH_2_)_5_COOH] and 0.069 g (0.31 μM) HCl*H-Pld-OMe. Yield, 0.301 g (85.5%), oily.

^1^H NMR (DMSO-d_6_, δ, ppm): −0.50—0.50 (m, 11H), 1.19–1.23 (6H, m); 1.37 (2H, m) 1.46 (2H, m); 1.91 (3H, s); 3.38 (2H, m); 3.61 (5H, m); 3.94 (m, 1H); 6.64 (br. s, 1H); 7.70–8.00 (m, 40H), 8.75 (br.s, 1H). ^13^C NMR (DMSO-d_6_, δ, ppm): 25.1, 26.2, 28.5, 31.6, 34.4, 42.2, 38.2, 51.6, 68.5, 117.6, 118.7, 131.0, 135.1, 135.9, 169.8, 175.6, 178.2. ^11^B {^1^H} NMR (DMSO-d_6_, δ, ppm): 5.8 (s, B(1)), −16.7 (s, B(2–6)), −18.1 (s, B(7–11)), −21.2 (s, B(12)). IR (KBr, cm^−1^): 2447 (ν(B-H)), 1720 ((ν(C=O)). Found, %: B 11.28, C 66.51, H 6.70, N 2.42. Calculated, %: B 11.45, C 66.79, H 6.76, N 2.47. ESI MS. Found, *m*/*z*: 794.75 {Ph_4_P^+^ + [B_12_H_11_O(CH_2_)_6_COPld]^2−^}. (C_39_H_56_B_12_N_2_O_5_P). Calculated: M = 793.57. Found, *m*/*z*: 625.60 {Ph_4_P^+^ + [B_12_H_11_O(CH_2_)_6_COOH]^2−^}. (C_31_H_44_B_12_O_3_P). Calculated: M = 625.38.

Sodium methyl-2-[2-(2-(2-carbonyl)amino]-3-(2-oxopyrrolidin-3-yl)propanoate)ethoxy)ethoxy]undecahydro-*closo*-dodecaborate Na_2_[B_12_H_11_O(CH_2_)_6_COPld-OMe] (Na_2_**5**). (Ph_4_P)_2_[B_12_H_11_O(CH_2_)_6_COPld] (0.227 g, 0.20 mmol) was dissolved in methanol (2 mL). A solution of Na[BPh_4_] (0.137 g, 0.40 mmol) in methanol (2 mL) was added, resulting in the formation of a white precipitate. The precipitate was removed by filtration. The filtrate was concentrated under reduced pressure to afford a white hygroscopic powder, which was dried under high vacuum. Yield, 0.049 g (49%).

## 4. Conclusions

This study successfully synthesized and evaluated a novel series of amino acid-functionalized *closo*-dodecaborate derivatives (Na_2_**2**–Na_2_**5**) for their antiviral activity against a contemporary, multidrug-resistant influenza A virus strain, A/Cheboksary/125/2020 (H1N1)pdm09.

The key findings and conclusions are as follows:

1. A clear structure–activity relationship was established. The nature of the amino acid residue attached to the boron cluster platform is a critical determinant of antiviral potency. The L-tryptophan-containing derivative Na_2_**2** demonstrated the most significant antiviral activity, achieving an IC_50_ value of 5.0 µg/mL. The L-histidine-based conjugate Na_2_**3** also exhibited notable though lower efficacy. In contrast, the methionine (Na_2_**4**) and lactam (Na_2_**5**) derivatives showed no significant activity within the tested concentration range, highlighting the importance of aromatic/heterocyclic residues for effective virus inhibition.
2. The observed activity of compounds Na_2_**2** and Na_2_**3** is particularly significant as it was demonstrated against a clinical influenza isolate with reduced susceptibility to neuraminidase inhibitors (oseltamivir, zanamivir) and full resistance to M2 ion channel blockers. This suggests that the boron cluster-amino acid conjugates possess a mechanism of action distinct from conventional anti-influenza drugs.
3. The compounds exhibited moderate cytotoxicity (CC_50_ = 80–160 µg/mL) and were noted to have lower aqueous solubility compared to their smaller *closo*-decaborate (B_10_) analogs. This reduced solubility may impact bioavailability and represents a key parameter for optimization in future drug design.
4. Extended evaluation against the A/IIV-Orenburg/83/2012 strain enabled direct comparison with reference compounds. Na_2_**2** exhibited significant antiviral effects comparable to the known lead B_10_-platform compound Na_2_[B_10_H_9_–O(CH_2_)_2_O(CH_2_)_3_C(O)–Trp–OCH_3_] and superior to rimantadine hydrochloride, confirming the viability of both boron cluster platforms for antiviral development.
5. The work confirms the *closo*-dodecaborate anion as a viable and promising inorganic platform for the development of antiviral agents, extending the concept previously established for *closo*-decaborate derivatives. The structural versatility of boron clusters enables fine-tuning of biological activity through rational spacer and pharmacophore selection.

In summary, this research underscores the potential of boron cluster-based hybrids as a novel strategy for combating drug-resistant influenza viruses. The identified structure–activity relationship provides a solid foundation for the rational design of next-generation antiviral agents with improved potency and pharmacological properties.

## Data Availability

Data are provided within the manuscript or Supplementary Information files.

## Supplementary Materials

The following supporting information can be downloaded at https://www.mdpi.com/article/10.3390/molecules30214225/s1, ^1^H NMR, ^13^C NMR, and ESI MS spectra of compounds (Ph_4_P)_2_**1**–(Ph_4_P)_2_**5**, SAR docking details.

## Abbreviations

The following abbreviations are used in this manuscript:

Trp	tryptophan
His	histidine
Pld	alanine-2-oxopyrrolidin-3-yl
Met	methionine
MDCK	Madin-Darby canine kidney
NMR	Nuclear magnetic resonance
CT_50_	50% cytotoxic concentration
NMM	N-methylmorpholine
IBCF	iso-butyl chloroformate
