# Supplementary material for: Exploring the Anti-Influenza Activity of closo-Borate Platforms: Structure–Activity Relationship of Amino Acid-Functionalized closo-Dodecaborate Derivatives Against Influenza Virus A/Cheboksary/125/2020 (H1N1)pdm09
Source: Molecules. 2025 Oct 29;30(21):4225. doi: 10.3390/molecules30214225 (PMC12610980; doi:10.3390/molecules30214225)
Supplement: Supplementary file 1 [file molecules-30-04225-s001.zip › molecules-3927015-supplementary.pdf]

Supplementary information

Exploring the Anti-Influenza Activity of *closo*-Borate Platforms: Structure–Activity Relationship of Amino Acid-Functionalized *closo*-Dodecaborate Derivatives Against Influenza Virus A/Cheboksary/125/2020 (H1N1)pdm09

Timur M. Garaev [1], Ilya I. Yudin [1], Natalya V. Breslav [1], Tatyana V. Grebennikova [1], Evgenii Yu. Matveev [2,3], Elizaveta A. Eshtukova-Shcheglova [3], Ilya E. Sokolov [4], Varvara V. Avdeeva [2,*], Konstantin Yu. Zhizhin [2,3] and Nikolai T. Kuznetsov [2]

[1] Gamaleya National Research Center for Epidemiology and Microbiology, Ministry of Health of Russian Federation, Moscow 123098, Russia; tmgaraev@gmail.com (T.M.G.); yudinilya2000@yandex.ru (I.I.Y.); n.belyakova1983@gmail.com (N.V.B.); t_grebennikova@mail.ru (T.V.G.)

[2] Kurnakov Institute of General and Inorganic Chemistry, Russian Academy of Sciences, Moscow 119991, Russia; cat1983@yandex.ru (E.Y.M.); zhizhin@igic.ras.ru (K.Y.Z.); ntkuz@igic.ras.ru (N.T.K.)

[3] Institute of Fine Chemical Technologies named after M. V. Lomonosov, MIREA—Russian Technological University, Moscow 119571, Russia; shchegs.ea@gmail.com

[4] Federal Research Centre of Nutrition and Biotechnology, Moscow 109240, Russia; sokolov.iliya1993@gmail.com

* Correspondence: avdeeva.varvara@mail.ru

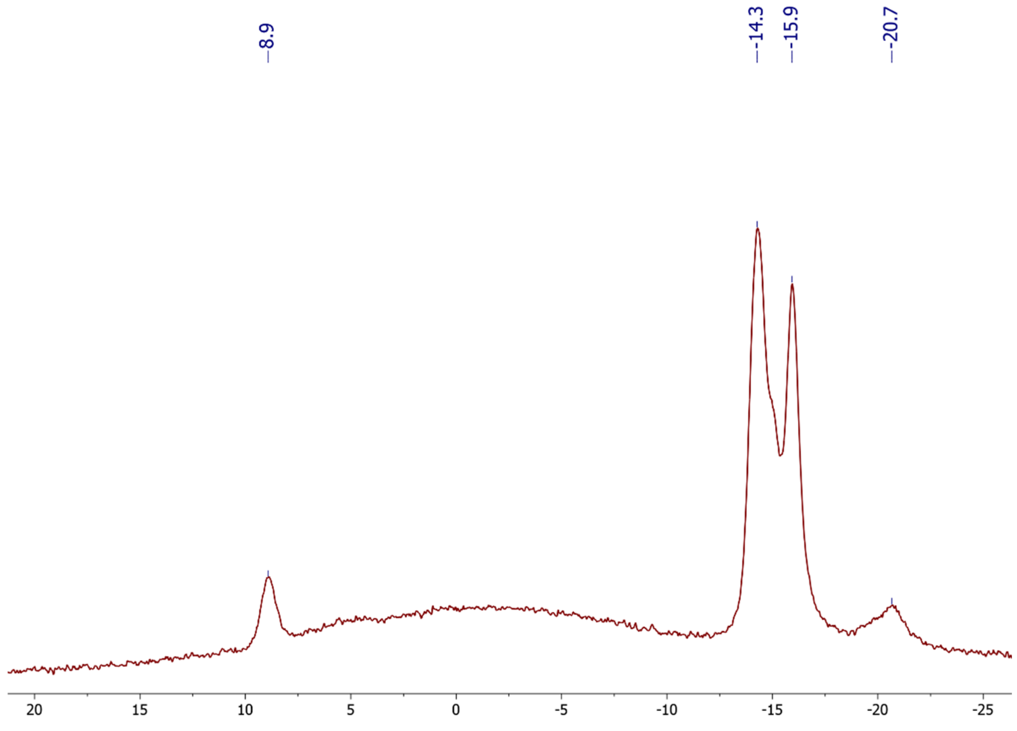

$^{11}B\{^{1}H\}$ NMR of **$(Ph_4P)_2$1**

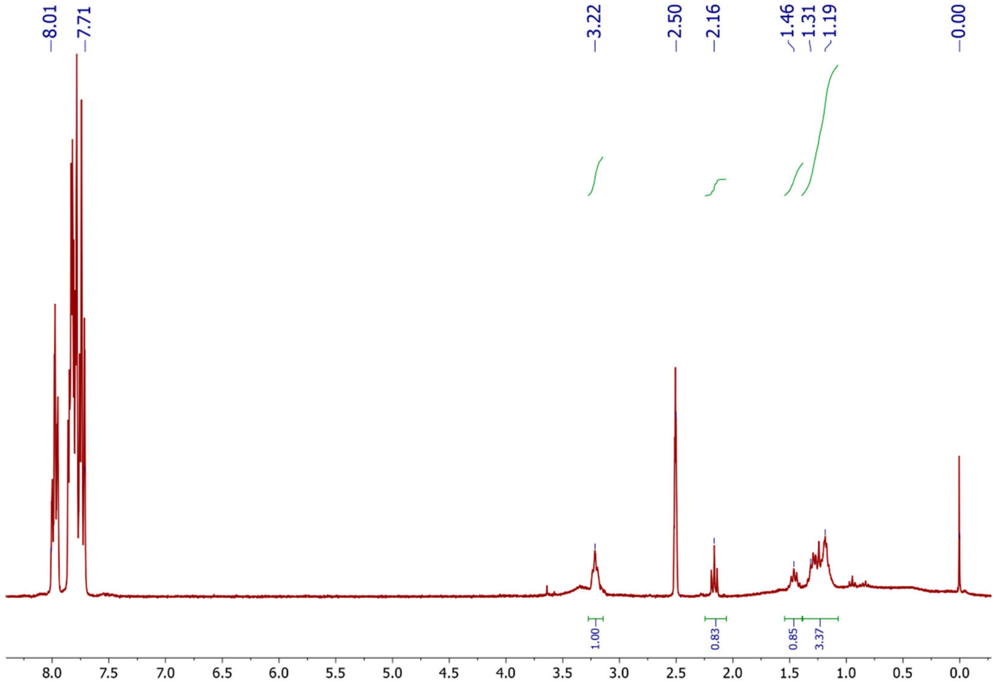

^{1}H NMR of **$(Ph_4P)_2$1**

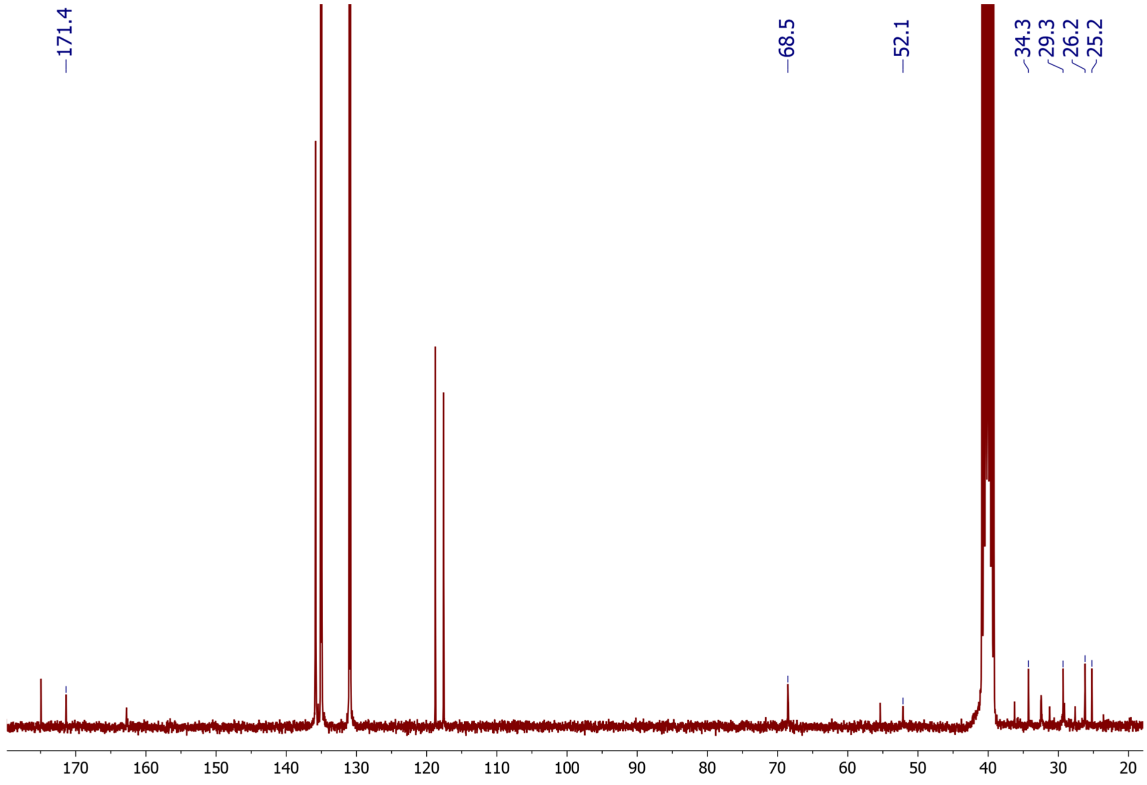

^{13}C NMR of **$(Ph_4P)_2$1**

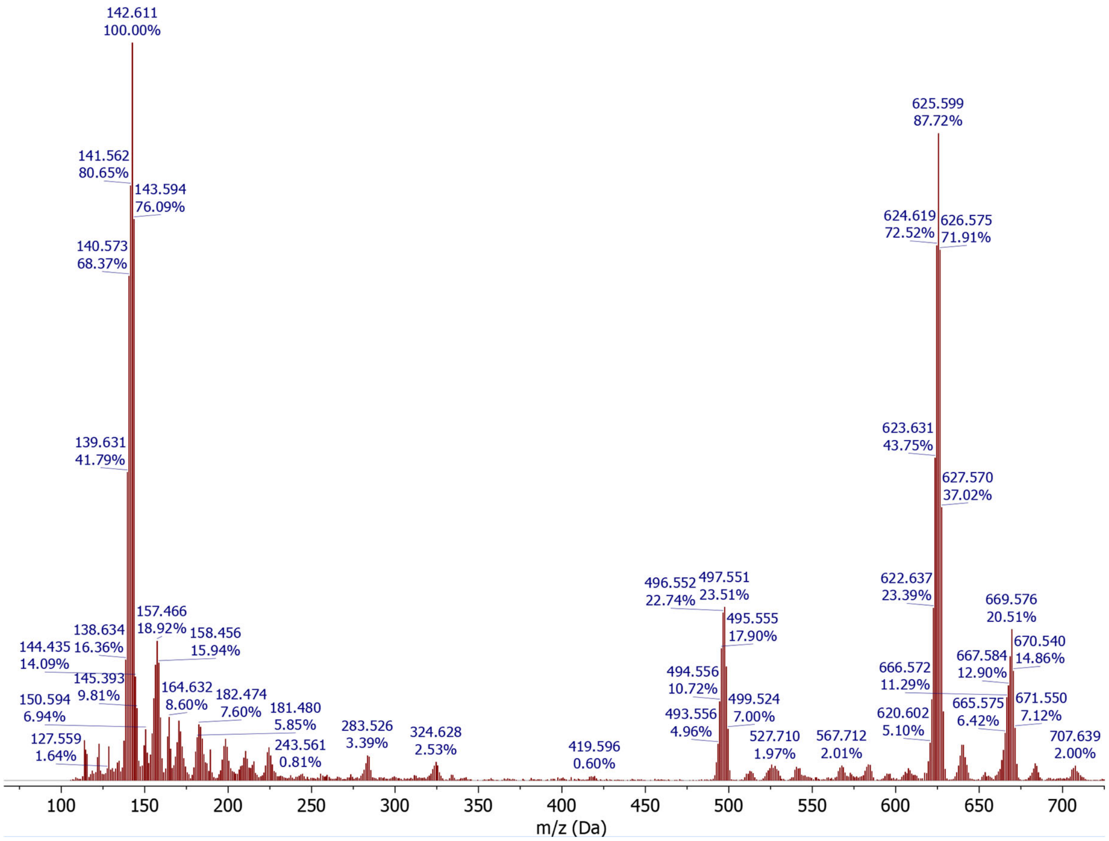

ESI MS of **$(Ph_4P)_2$1**

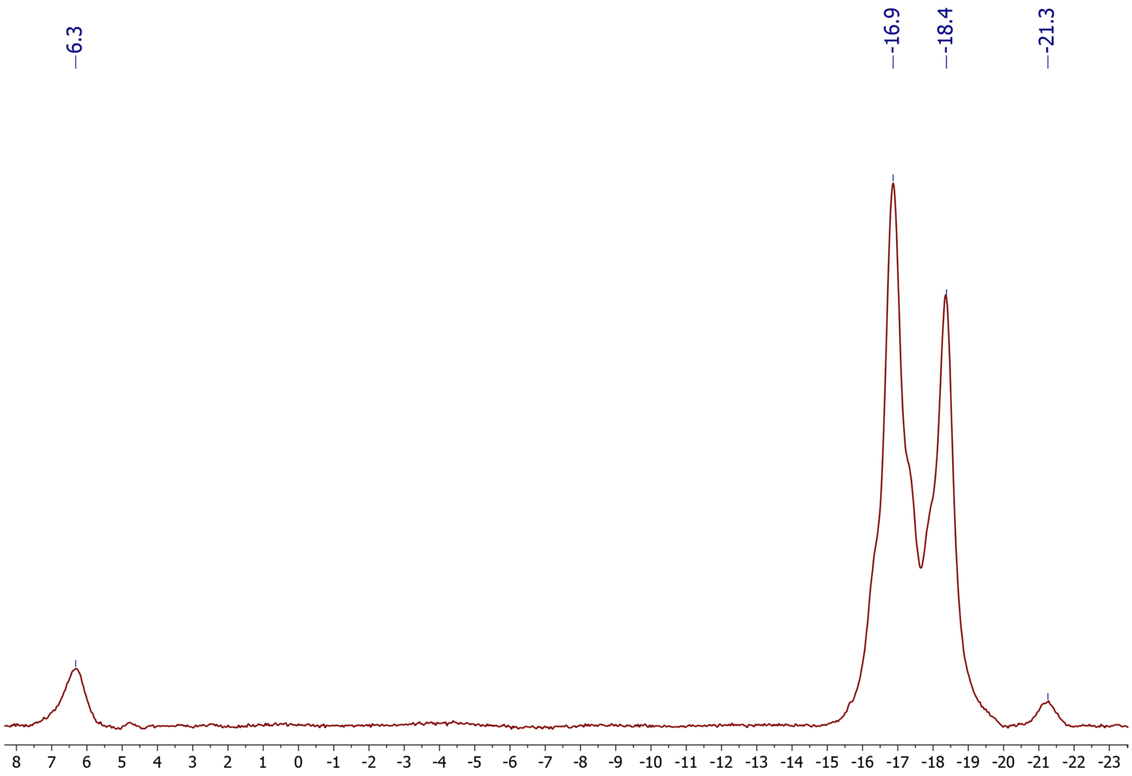

$^{11}B\{^1H\}$ NMR of **$(Ph_4P)_2$2**

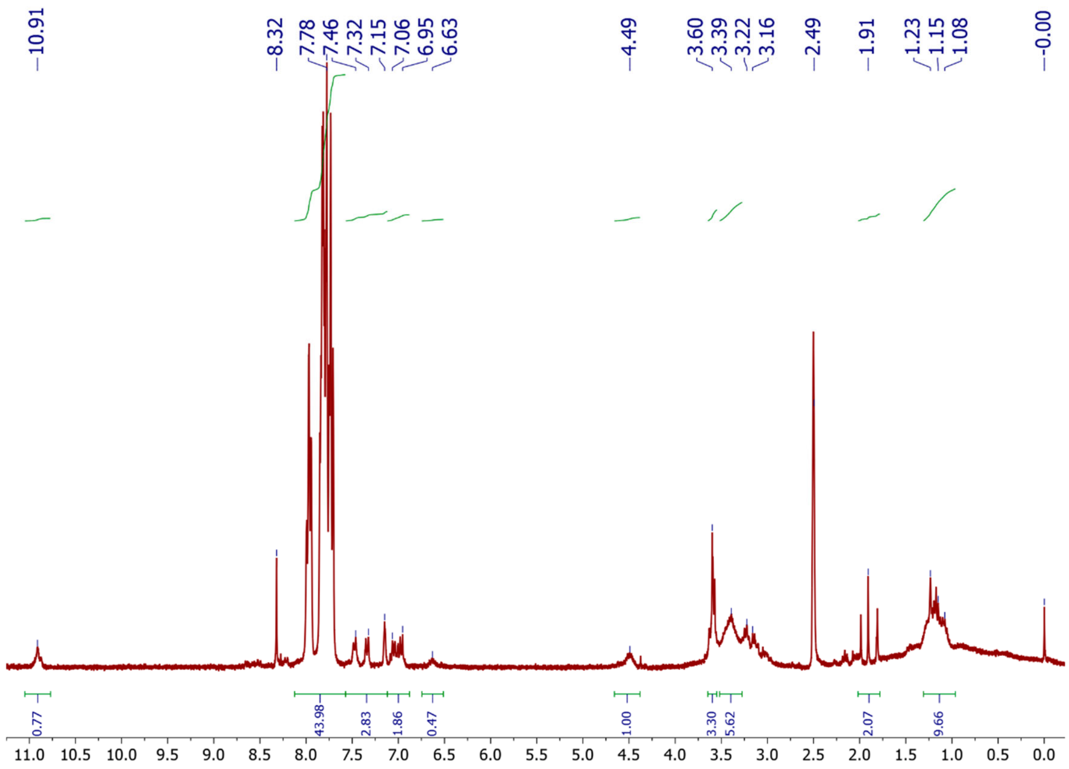

^{1}H NMR of **$(Ph_4P)_2$2**

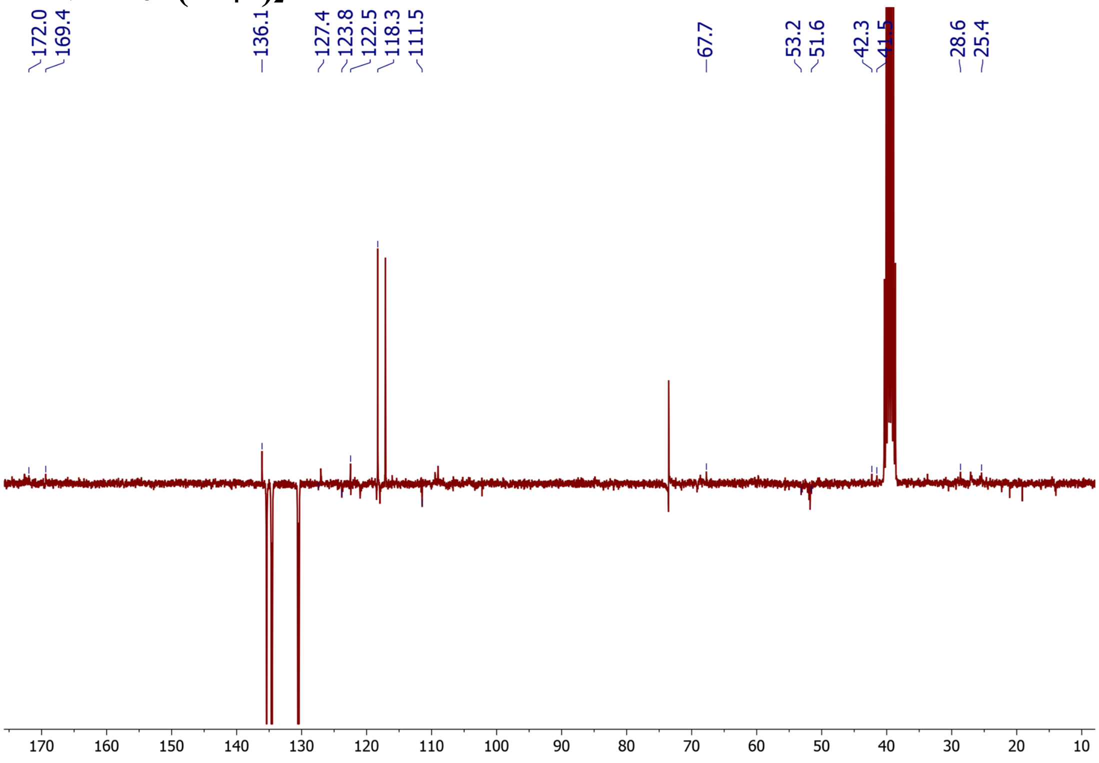

^{13}C NMR of **$(Ph_4P)_2$2**

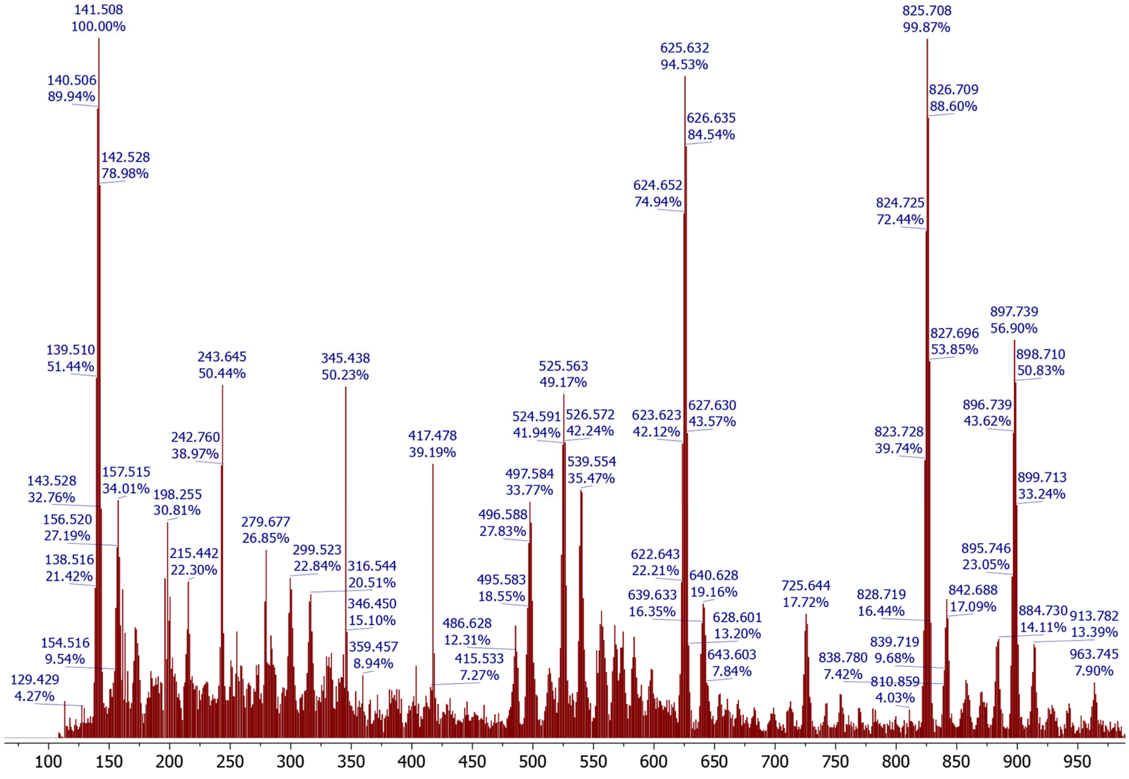

ESI MS of **$(Ph_4P)_2$2**

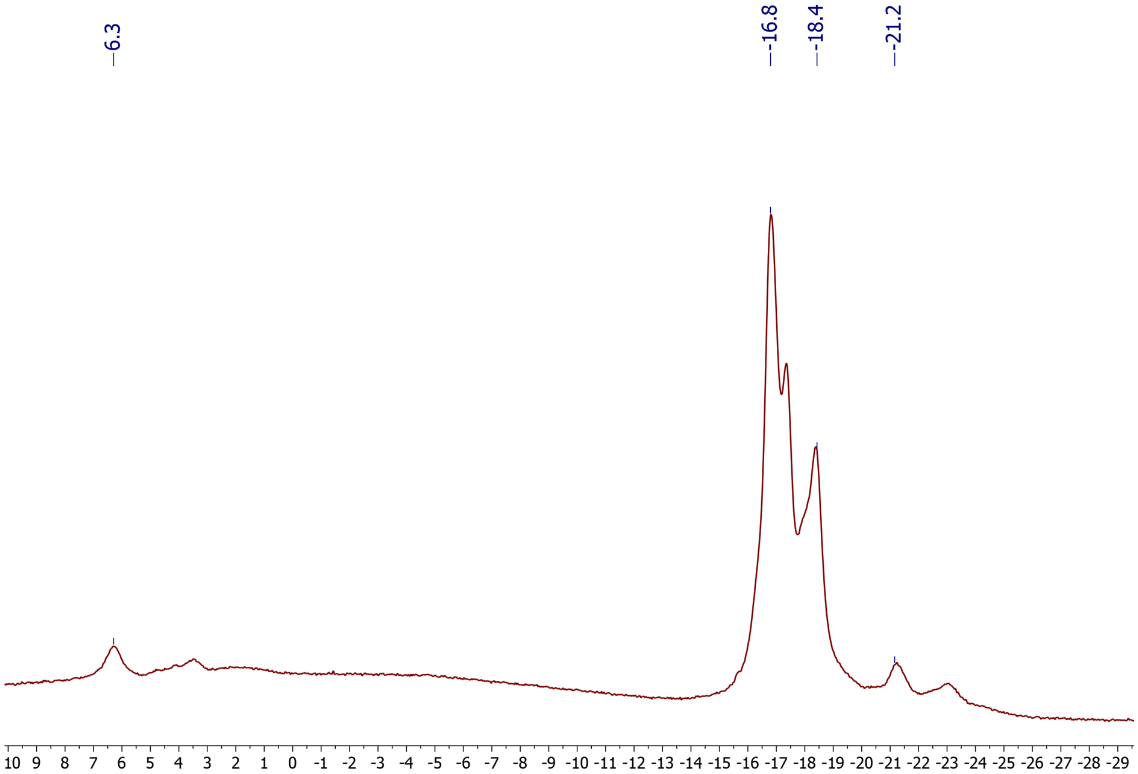

$^{11}B\{^1H\}$ NMR of **$(Ph_4P)_2$3**

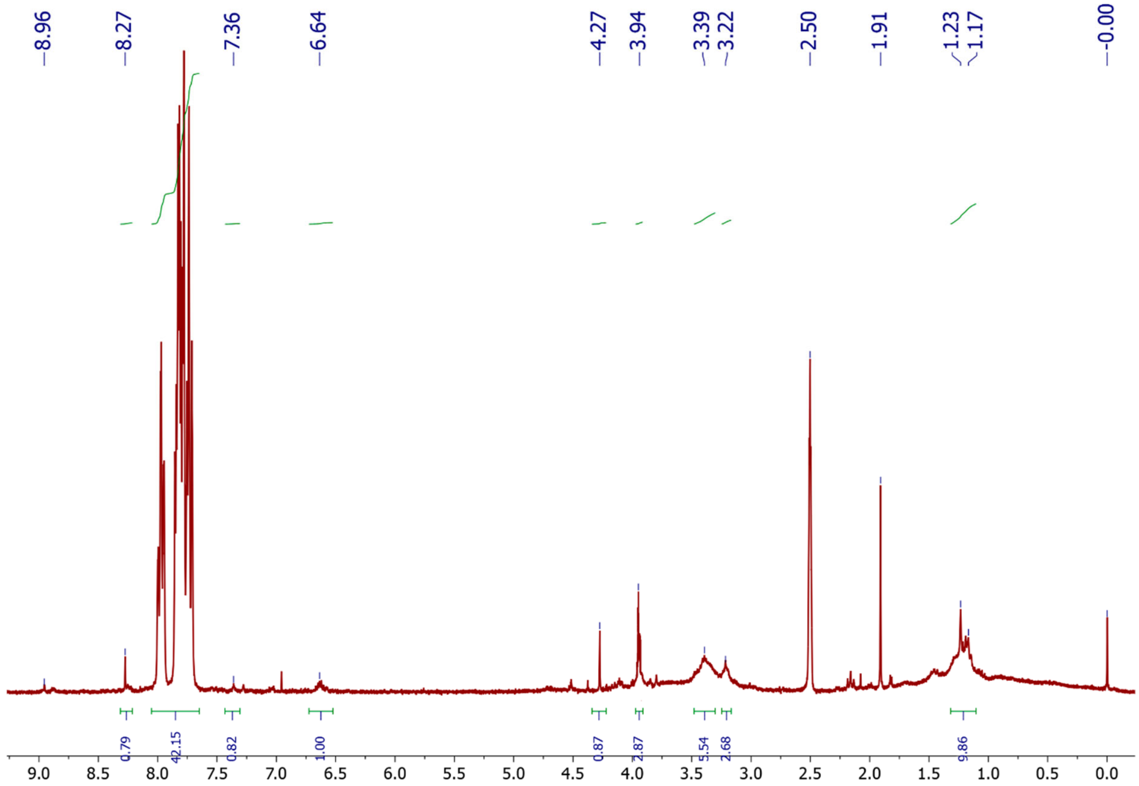

^{1}H NMR of **$(Ph_4P)_2$3**

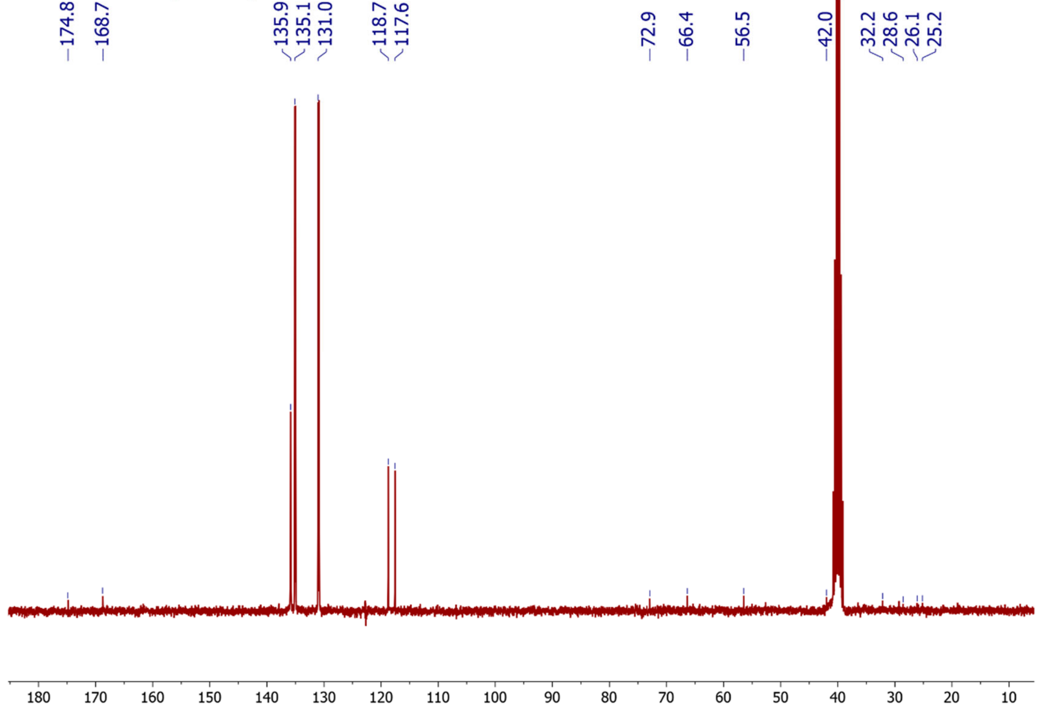

^{13}C NMR of **$(Ph_4P)_2$3**

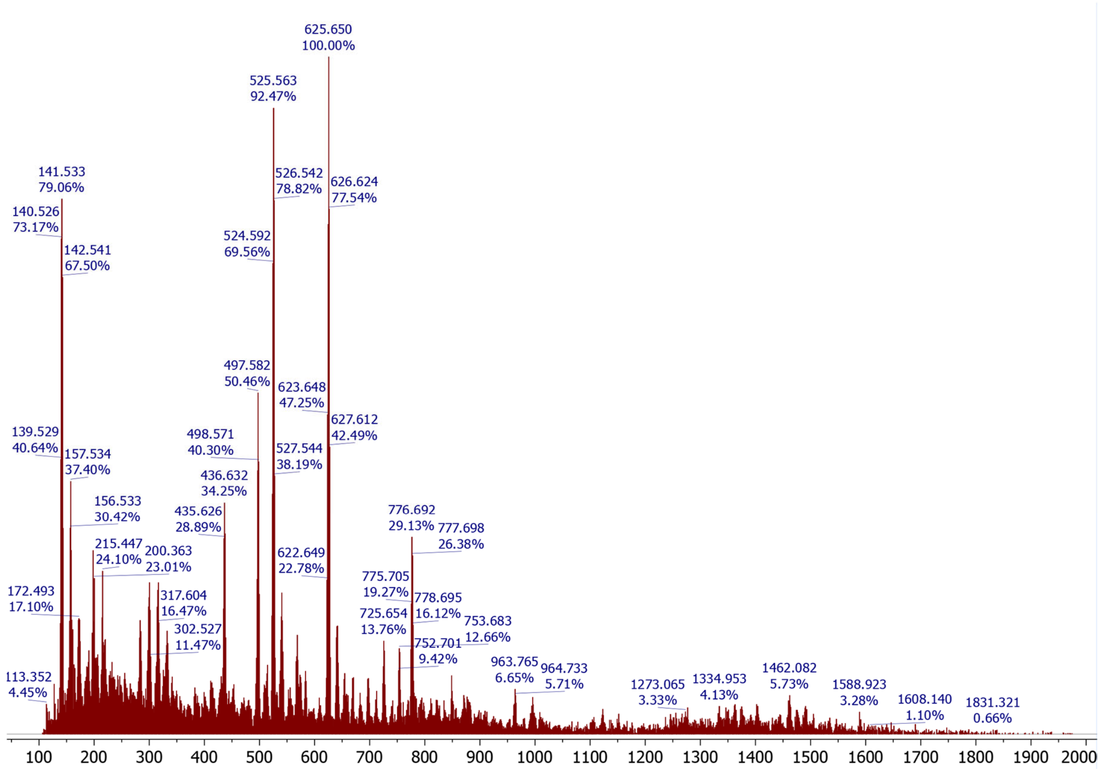

ESI MS of **(Ph$_4$P)$_2$3**

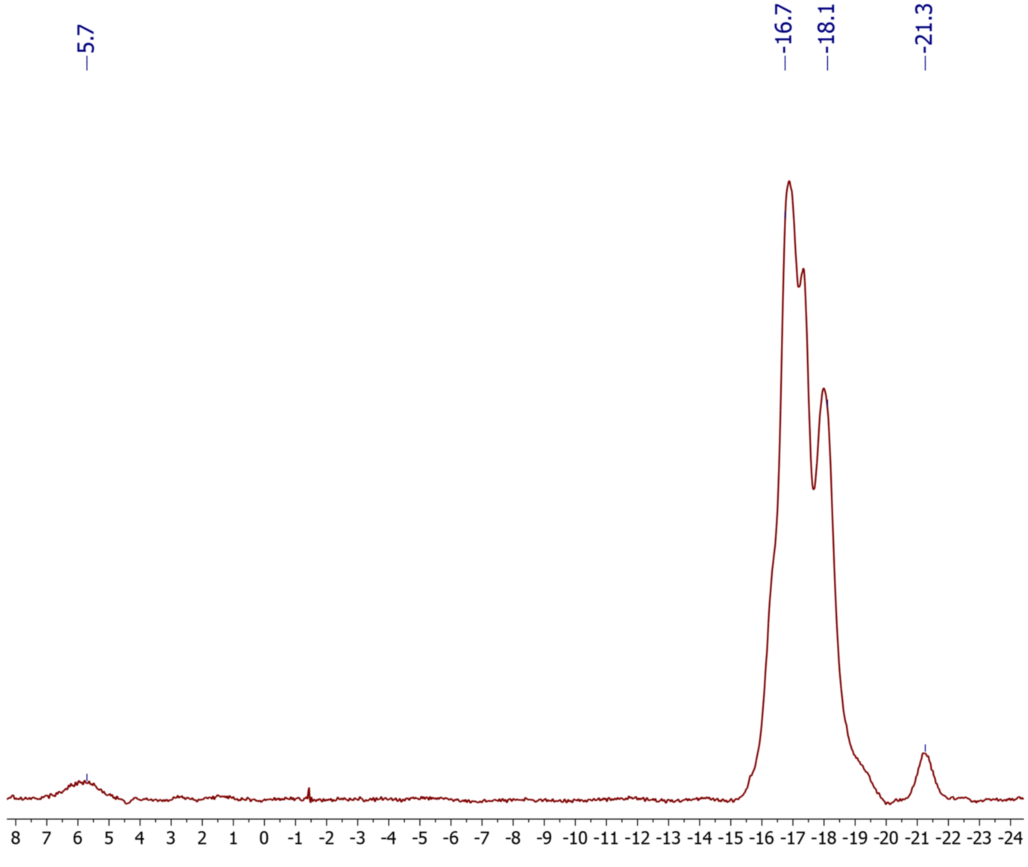

^{11}B{^{1}H} NMR of **(Ph$_4$P)$_2$4**

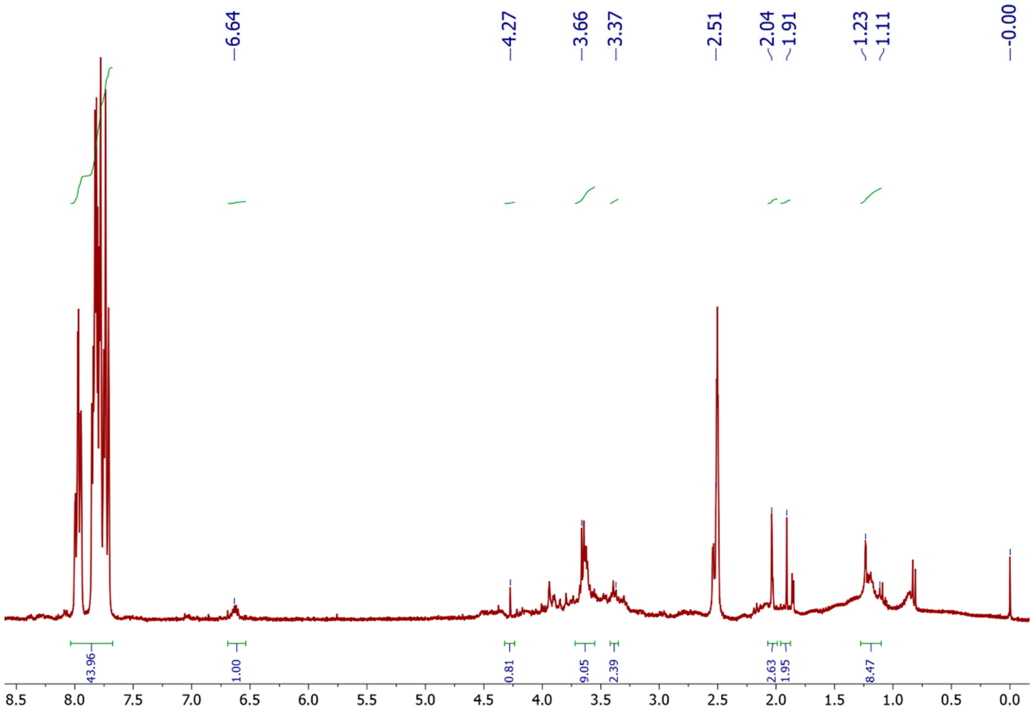

^{1}H NMR of **$(Ph_4P)_2$4**

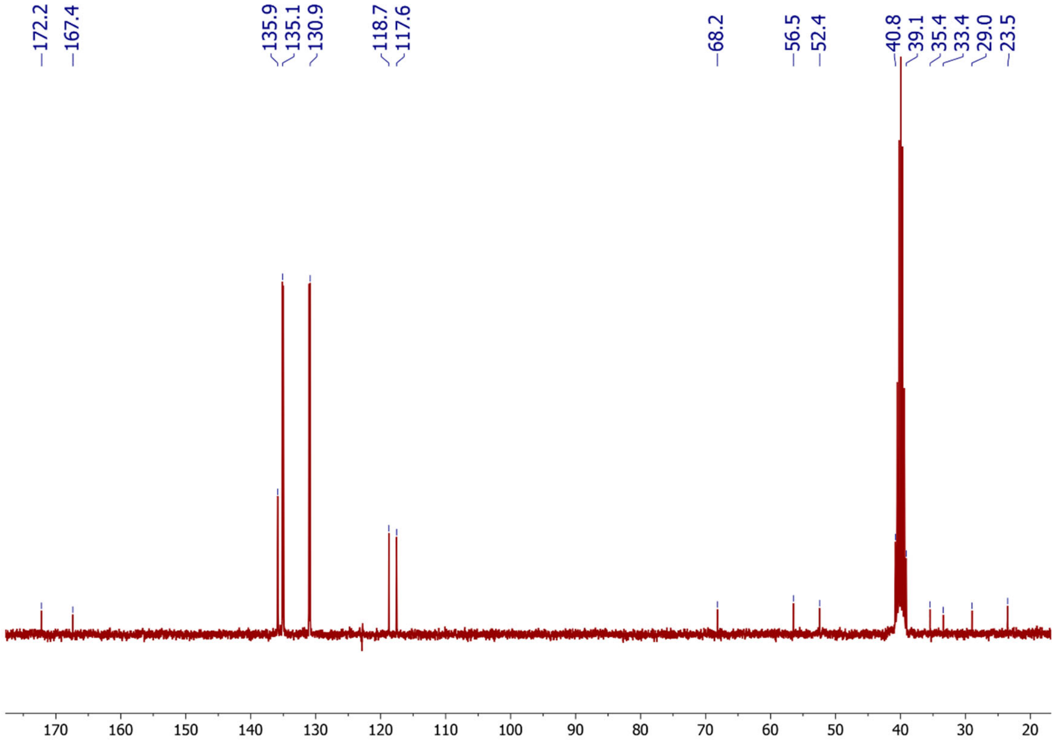

^{13}C NMR of **$(Ph_4P)_2$4**

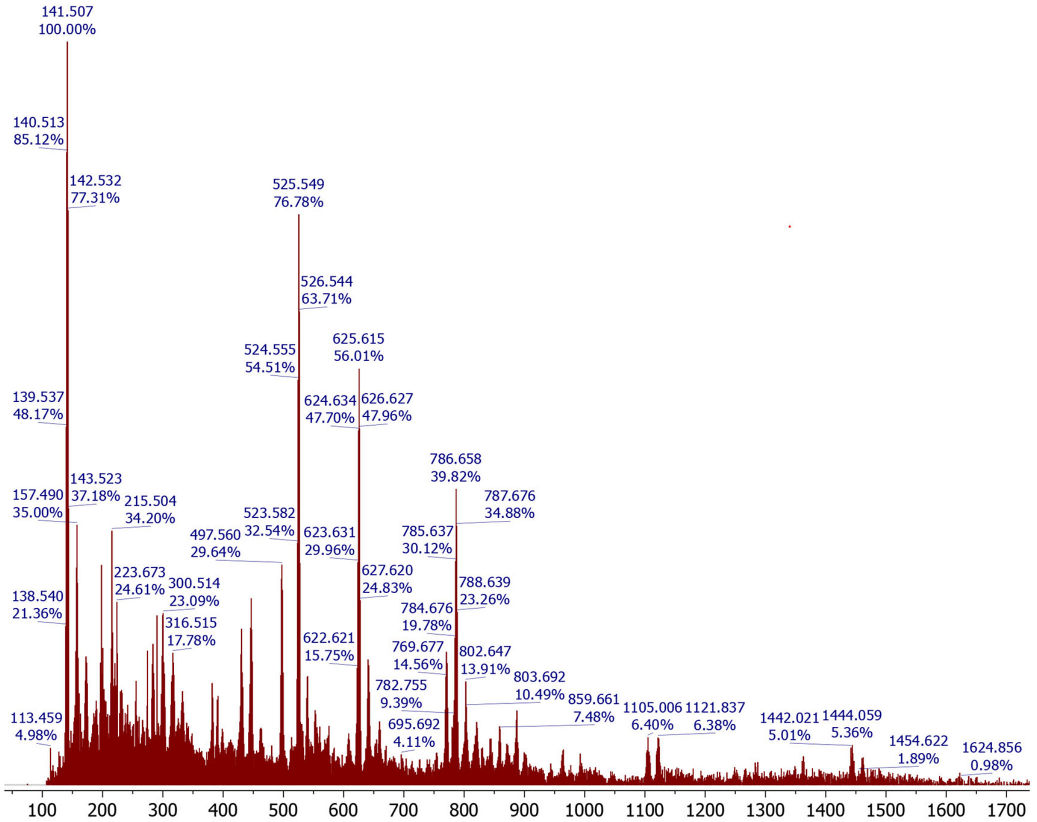

ESI MS of **(Ph$_4$P)$_2$4**

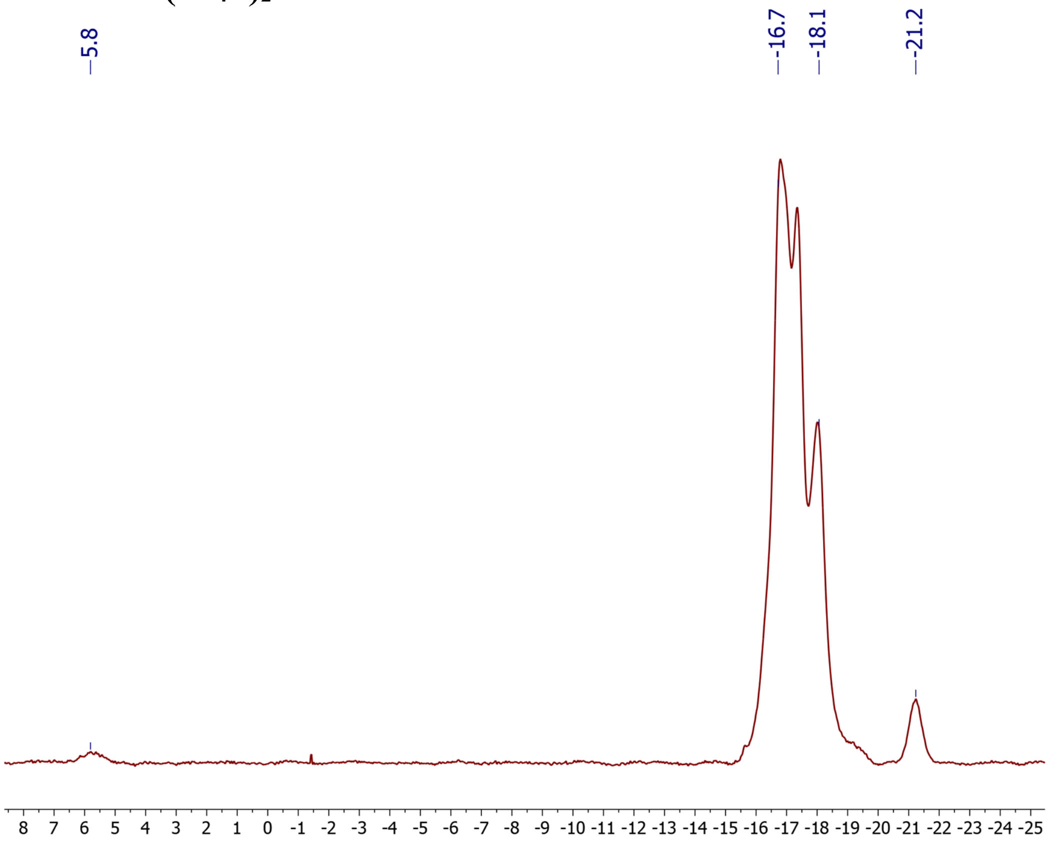

$^{11}B\{^{1}H\}$ NMR of **$(Ph_4P)_2$5**

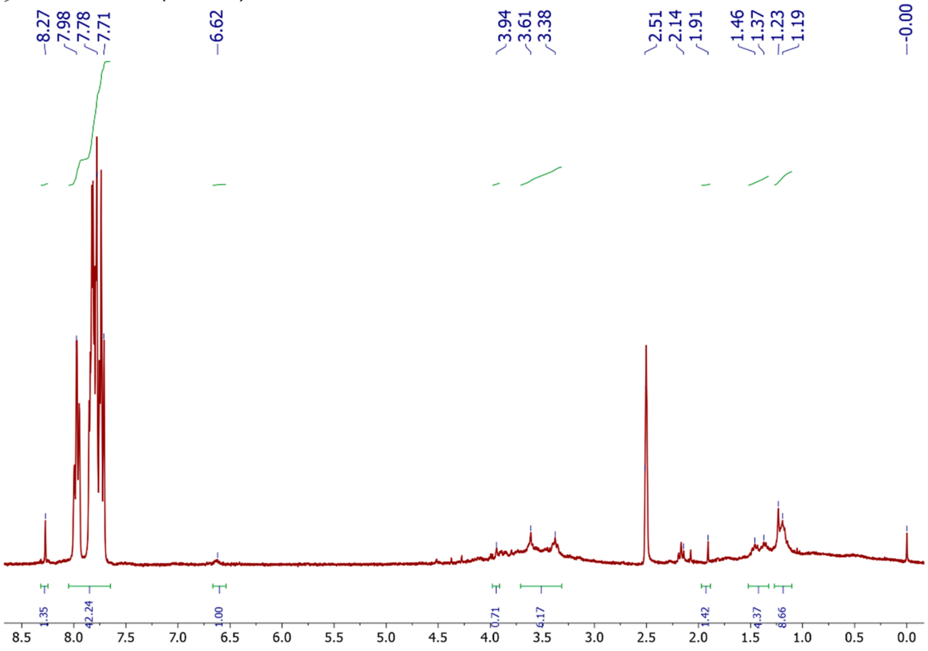

^{1}H NMR of **$(Ph_4P)_2$5**

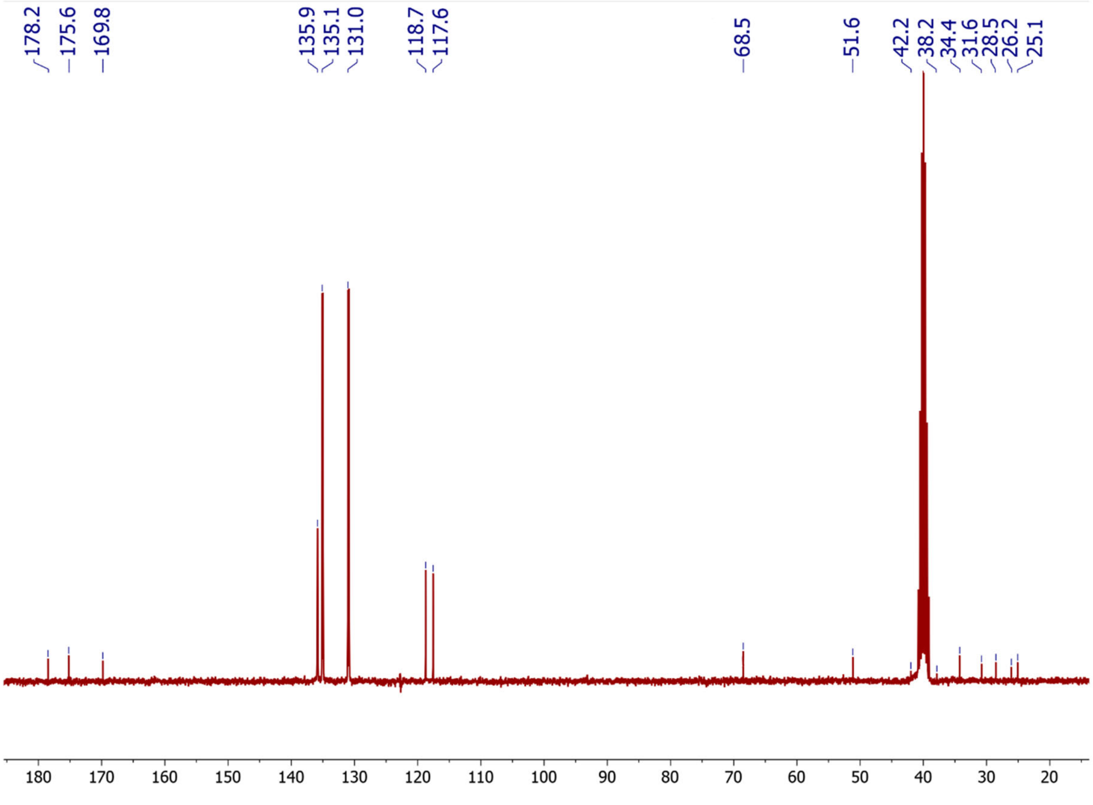

^{13}C NMR of **$(Ph_4P)_2$5**

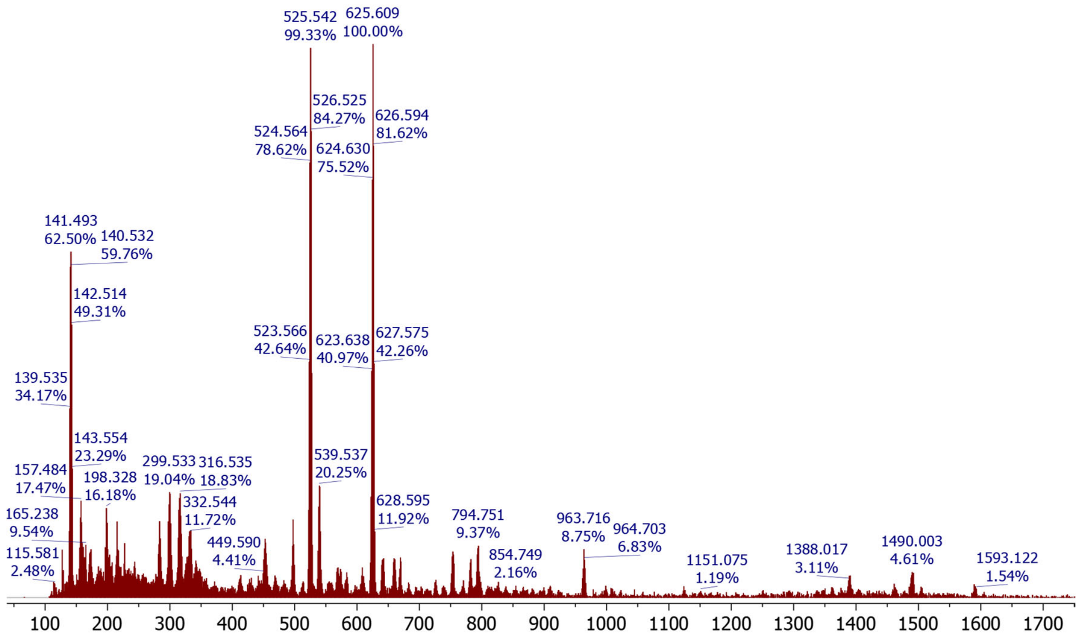

ESI MS of **$(Ph_4P)_2$5**

>A/IIV-Orenburg/83/2012|M2|EPI_ISL_1360030

transmembrane domain of M2

MSLLTEVETPTRSEWECRCSDS [SDPLVIAANIIGILHLILWITDRLFFKCIYRRFKYGLK] RGPSTEG
VPESMREEYQQEQQSAVDVDDGHFVNIELE

>A/Human/Cheboksary/125/2020(H1N1)|M2|EPI_ISL_483726

transmembrane domain of M2

MSLLTEVETPTRSEWECRCSGS [NDPLVIAANIIGILHLILWITDRLFFKCIYRRFKYGLK] RGPSTEG
VPESMREEYQQEQQSAVDVDDGHFVNIELE

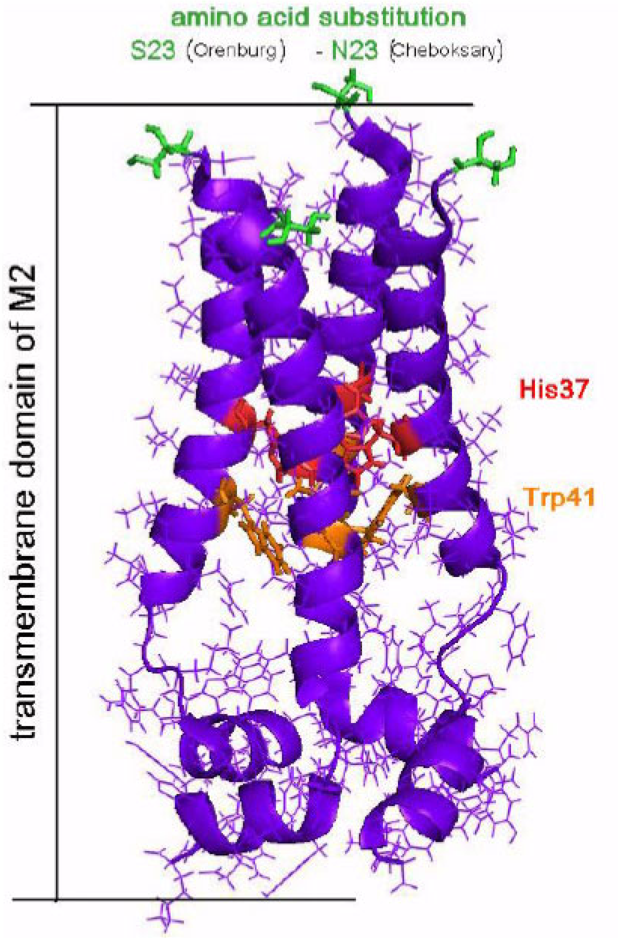

Figure. S1. The M2 gene sequences (A/IIV-Orenburg/83/2012|M2|EPI_ISL_1360030 and A/Human/Cheboksary/125/2020(H1N1)|M2|EPI_ISL_483726) were isolated and characterized at the Laboratory of Influenza Etiology and Epidemiology of the N.F. Gamaleya National Research Center for Epidemiology and Microbiology. The transmembrane domain (TM-domain) is highlighted with square brackets. The His37 tetrad (*red*) and Trp41 (*orange*) play crucial roles in proton conductance activation. A S23N substitution (*green*) was identified in the TM-domain entrance group. The cartoon representation depicts the TM M2 model with key amino acid residues indicated.

Table S1. Assessment of ADMET chemical properties for compounds Na_2**2** and $Na_2[B_{10}H_9–O(CH_2)_2O(CH_2)_3C(O)–Trp–OCH_3]$ in the admetSAR online service

Compound 2Na[$B_{12}H_{11}$-THP-Trp-OMe] ($Na_2$2)

ADMET Predicted Profile --- Classification

Model	Result	Probability
**Absorption**		
Blood-Brain Barrier	BBB+	0.9505
Human Intestinal Absorption	HIA+	0.9905
Caco-2 Permeability	Caco2-	0.6648
P-glycoprotein Substrate	Substrate	0.5660
P-glycoprotein Inhibitor	Non-inhibitor	0.8011
	Inhibitor	0.6824
Renal Organic Cation Transporter	Non-inhibitor	0.8072
**Distribution**		
Subcellular localization	Mitochondria	0.7086
**Metabolism**		
CYP450 2C9 Substrate	Non-substrate	0.7736
CYP450 2D6 Substrate	Non-substrate	0.8418
CYP450 3A4 Substrate	Substrate	0.5139
CYP450 1A2 Inhibitor	Non-inhibitor	0.6186
CYP450 2C9 Inhibitor	Non-inhibitor	0.7340
CYP450 2D6 Inhibitor	Non-inhibitor	0.8909
CYP450 2C19 Inhibitor	Non-inhibitor	0.7049
CYP450 3A4 Inhibitor	Inhibitor	0.5086
CYP Inhibitory Promiscuity	High CYP Inhibitory Promiscuity	0.8589
**Excretion**		
**Toxicity**		
Human Ether-a-go-go-Related Gene Inhibition	Weak inhibitor	0.9961
	Non-inhibitor	0.7852
AMES Toxicity	Non AMES toxic	0.8492
Carcinogens	Non-carcinogens	0.9361
Fish Toxicity	High FHMT	0.7578
Tetrahymena Pyriformis Toxicity	High TPT	0.8961
Honey Bee Toxicity	Low HBT	0.7282
Biodegradation	Not ready biodegradable	0.9953
Acute Oral Toxicity	III	0.5899
Carcinogenicity (Three-class)	Non-required	0.6273

ADMET Predicted Profile --- Regression

Model	Value	Unit
**Absorption**		
Aqueous solubility	-3.3285	LogS
Caco-2 Permeability	0.4415	LogPapp, cm/s
**Distribution**		
**Metabolism**		
**Excretion**		
**Toxicity**		

Rat Acute Toxicity	2.3195	LD50, mol/kg
Fish Toxicity	1.4248	pLC50, mg/L
Tetrahymena Pyriformis Toxicity	0.3422	pIGC50, ug/L

Compound $Na_2[B_{10}H_9–O(CH_2)_2O(CH_2)_3C(O)–Trp–OCH_3]$

Results

ADMET Predicted Profile --- Classification

Model	Result	Probability
**Absorption**		
Blood-Brain Barrier	BBB-	0.8859
Human Intestinal Absorption	HIA-	0.7337
Caco-2 Permeability	Caco2-	0.6503
P-glycoprotein Substrate	Substrate	0.8001
P-glycoprotein Inhibitor	Non-inhibitor	0.7355
	Non-inhibitor	0.9942
Renal Organic Cation Transporter	Non-inhibitor	0.8397
**Distribution**		
Subcellular localization	Mitochondria	0.6998
**Metabolism**		
CYP450 2C9 Substrate	Non-substrate	0.7777
CYP450 2D6 Substrate	Non-substrate	0.7955
CYP450 3A4 Substrate	Substrate	0.6354
CYP450 1A2 Inhibitor	Non-inhibitor	0.7334
CYP450 2C9 Inhibitor	Non-inhibitor	0.7729
CYP450 2D6 Inhibitor	Non-inhibitor	0.8623
CYP450 2C19 Inhibitor	Non-inhibitor	0.7492
CYP450 3A4 Inhibitor	Non-inhibitor	0.8324
CYP Inhibitory Promiscuity	Low CYP Inhibitory Promiscuity	0.9339
**Excretion**		
**Toxicity**		
Human Ether-a-go-go-Related Gene Inhibition	Weak inhibitor	0.9709
	Inhibitor	0.5142
AMES Toxicity	Non AMES toxic	0.5737
Carcinogens	Non-carcinogens	0.9394
Fish Toxicity	High FHMT	0.8218
Tetrahymena Pyriformis Toxicity	High TPT	0.9935
Honey Bee Toxicity	Low HBT	0.7156
Biodegradation	Not ready biodegradable	0.9949
Acute Oral Toxicity	III	0.5824
Carcinogenicity (Three-class)	Non-required	0.5609

ADMET Predicted Profile --- Regression

Model	Value	Unit
**Absorption**		
Aqueous solubility	-2.8189	LogS
Caco-2 Permeability	-0.2584	LogPapp, cm/s

Distribution		
Metabolism		
Excretion		
Toxicity		
Rat Acute Toxicity	2.6274	LD50, mol/kg
Fish Toxicity	1.5354	pLC50, mg/L
Tetrahymena Pyriformis Toxicity	0.5270	pIGC50, ug/L
